# Supplementary material for: Chloroplast transformation for bioencapsulation and oral delivery using the immunoglobulin G fragment crystallizable (Fc) domain
Source: Sci Rep. 2023 Nov 2;13:18916. doi: 10.1038/s41598-023-45698-9 (PMC10622566; doi:10.1038/s41598-023-45698-9)
Supplement: Supplementary file 1 — Supplementary Information. [file 41598_2023_45698_MOESM1_ESM.pdf]

Online Supplementary Information

Chloroplast transformation for bioencapsulation and oral delivery using the immunoglobulin G fragment crystallizable (Fc) domain

Lisa LaManna[1], Chih-Hsuan Chou[3], Hanqin Lei[3], Elisabeth R. Barton[3*], and Pal Maliga[1,2*]

[1] Waksman Institute of Microbiology, Rutgers University, Piscataway, NJ 08854, USA. [2] Department of Plant Biology, Rutgers University, New Brunswick, NJ 08901, USA. [3] Department of Applied Physiology & Kinesiology, University of Florida, College of Health and Human Performance, Gainesville, FL, 32611, USA

***Correspondence and requests for biomaterials should be addressed to: maliga@waksman.rutgers.edu**

***Corresponding authors: erbarton@ufl.edu maliga@waksman.rutgers.edu**

Supplementary Information

Supplementary Figure S1. Tissue culture assay to test expression of spectinomcyin resistance in tobacco seedlings.

Supplementary Figure S2. Marker excision in the TVV plants.

Supplementary Figure S3. Annotated SnapGene files of TVV vectors.

Supplementary Figure S4. Original Southern blot corresponding to Fig 1b, upper.

Supplementary Figure S5. Original Southern blot corresponding to Fig 1b, lower.

Supplementary Figure S6. The original Northern blot corresponding to Fig 1c, upper.

Supplementary Figure S7. Original agarose gel electrophoresis of RNA extracts used to generate the Northern blots in Fig 1c.

Supplementary Figure S8. Original Northern blot corresponding to Fig 1c, lower.

Supplementary Figure S9. Original Coomassie brilliant blue gel corresponding to Fig 2a.

Supplementary Figure S10. Four replicates of the original immunoblots used to calculate the accumulation of GFP-Fc1 in TVV2 lines.

Supplementary Figure S11. Four replicates of the original immunoblots used to calculate the accumulation of proIGF1-Fc1NC and proIGF1-Fc1C proteins in TVV3 and TVV4 lines.

Supplementary Figure S12. Four replicates of the original immunoblots used to detect protein accumulation in the leaves of two TVV2 lines.

Supplementary Figure S13. Four replicates of the original immunoblots used to detect protein accumulation in the leaves of TVV3 lines.

Supplementary Figure S14. Four replicates of the original immunoblots used to detect protein accumulation in the leaves of TVV4 lines.

Supplementary Figure S15. Original Coomassie brilliant blue gel corresponding to Fig 3f.

Supplementary Figure S16. Two replicates of the original immunoblots used to detect the forms of the Fc fusions post lyophilization in each line.

Supplementary Table S1. DNA sequence of TVV plastid transformation vectors.

Seed assay to testing expression of *aadA* in progeny. Germinating seedlings on a medium containing spectinomycin is an assay to confirm uniform transformation of plastid genomes. We germinated seedlings of each of the transplastomic lines and found that they exhibited poor spectinomycin resistance: the seedlings were pale on 200 and 500 mg/L when germinated on RMOP medium containing 3% sucrose, the medium we normally use to test spectinomycin resistance on spectinomycin sulphate. The only exception was Nt-pMRR13, a line in which *aadA* is expressed from a P*rrn*L*atpB* 5' regulatory region, and only on 200 mg/L spectinomcyin . All other constructs had P*rbcL*, a *rbcL* gene promoter and leader. When the same seeds were germinated on an RMOP medium without sucrose, the seedlings were uniformly resistant, indicated by their green color (Supplemental Figure S1). The plastid *rbcL* gene is a photosynthetic gene. We surmise that the translation of its mRNA is susceptible to suppression by sucrose in the culture medium. In contrast, when the seed was germinated in the absence of sucrose in the culture medium, the seedlings were uniformly green indicating normal development of the photosynthetic machinery due to uninhibited protein synthesis on plastid ribosomes.

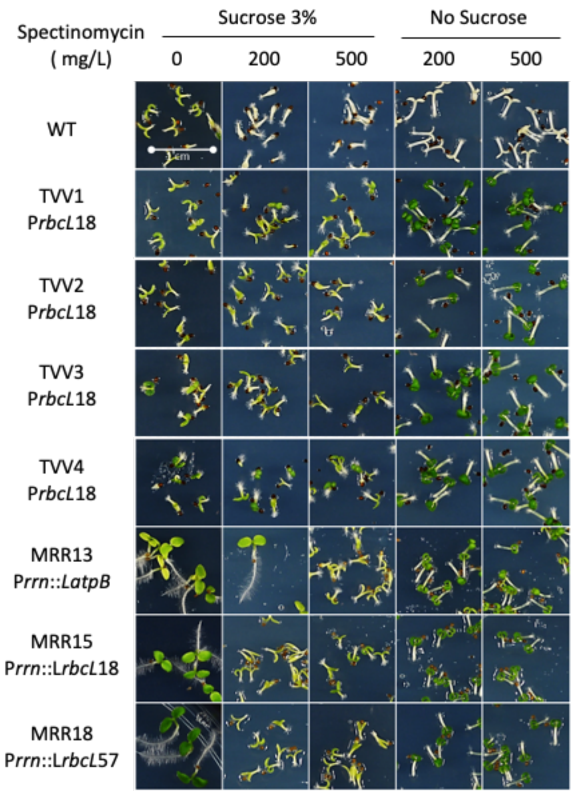

Supplementary Figure S1. Tissue culture assay to test expression of spectinomcyin resistance in tobacco seedlings. Note that uniform, dark green color of seedlings in the absence of sucrose.

Excision of the plastid marker gene by the Int site-specific recombinase.
Marker gene excision was accomplished by crossing T1 progeny as maternal parent with Nt-pYZ pollen parents, which were transformed with an Agrobacterium pKO117 binary vector to excise the *aadA* marker gene. Vector Nt-KO117 carries a nuclear encoded and chloroplast-targeted PhiC31 phage site-specific integrase (Lutz et al., 2004). Excision of *aadA* in the pYZ14, pYZ15 and pYZ16 plants was complete (Zhang et al., 2022). PCR analysis using a forward primer located in the T*rnP* gene and a reverse primer located in the 3'rps12/7 promoter of the chloroplast genome was used to track excision. Loss of the selectable marker was evident in the F1 generation and advanced in the F2 generation. Interestingly, most of the F1 progeny derived from the cross was spectinomycin resistant indicated by the green color of most seedlings (Supplemental Fig. S2a) and the 1.5 kb PCR product (Supplementary Fig. S2b). In the F2 marker-free plants were obtained (Supplementary Fig. S2b).

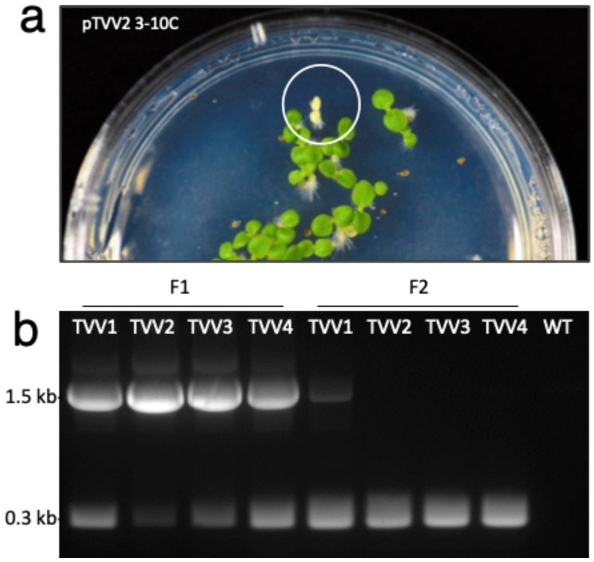

Supplementary Figure S2. Marker excision in the TVV plants. (**a**) Spectinomycin resistance of most seedlings in the F1 progeny indicates that excision of the *aadA* marker gene is incomplete. (**b**) PCR amplification indicates presence of *aadA* in most seedlings in F1, and absence of *aadA* in F2. PCR analysis using a forward primer located in the T*rnP* gene and a reverse primer located in the 3'rps12/7 promoter was used to track excision (Table 3).

References

Lutz KA, Corneille S, Azhagiri AK, Svab Z, Maliga P (2004) A novel approach to plastid transformation utilizes the phiC31 phage integrase. Plant J **37:** 906-913

Zhang Y, Ananyev G, Matsuoka A, Dismukes GC, Maliga P (2022) Cyanobacterial Photosystem II reaction center design in tobacco chloroplasts increases biomass in low light. Plant Physiology**:** kiac578

Supplementary Figure 3A

Unique Cutters **Bold**

SapI
BspQI

5′ CTTCCGCTTCCTCGCTCACTGACTCGCTGCGCTCGGTCGTTCGGCTGCGGCGAGCGGTATCAGCT 65
3′ GAAGGCGAAGGAGCGAGTGACTGAGCGACGCGAGCCAGCAAGCCGACGCCGCTCGCCATAGTCGA

CACTCAAAGGCGGTAATACGGTTATCCACAGAATCAGGGGATAACGCAGGAAAGAACATGTGAGC 130
GTGAGTTTCCGCCATTATGCCAATAGGTGTCTTAGTCCCCTATTGCGTCCTTTCTTGTACACTCG

AAAAGGCCAGCAAAAGGCCAGGAACCGTAAAAAGGCCGCGTTGCTGGCGTTTTTCCATAGGCTCC 195
TTTTCCGGTCGTTTTCCGGTCCTTGGCATTTTTCCGGCGCAACGACCGCAAAAAGGTATCCGAGG
ori

GCCCCCCTGACGAGCATCACAAAAATCGACGCTCAAGTCAGAGGTGGCGAAACCCGACAGGACTA 260
CGGGGGGACTGCTCGTAGTGTTTTTAGCTGCGAGTTCAGTCTCCACCGCTTTGGGCTGTCCTGAT
ori

TAAAGATACCAGGCGTTTCCCCCTGGAAGCTCCCTCGTGCGCTCTCCTGTTCCGACCCTGCCGCT 325
ATTTCTATGGTCCGCAAAGGGGGACCTTCGAGGGAGCACGCGAGAGGACAAGGCTGGGACGGCGA
ori

TACCGGATACCTGTCCGCCTTTCTCCCTTCGGGAAGCGTGGCGCTTTCTCATAGCTCACGCTGTA 390
ATGGCCTATGGACAGGCGGAAAGAGGGAAGCCCTTCGCACCGCGAAAGAGTATCGAGTGCGACAT
ori

GGTATCTCAGTTCGGTGTAGGTCGTTCGCTCCAAGCTGGGCTGTGTGCACGAACCCCCCGTTCAG 455
CCATAGAGTCAAGCCACATCCAGCAAGCGAGGTTCGACCCGACACACGTGCTTGGGGGGCAAGTC
ori

CCCGACCGCTGCGCCTTATCCGGTAACTATCGTCTTGAGTCCAACCCGGTAAGACACGACTTATC 520
GGGCTGGCGACGCGGAATAGGCCATTGATAGCAGAACTCAGGTTGGGCCATTCTGTGCTGAATAG
ori

GCCACTGGCAGCAGCCACTGGTAACAGGATTAGCAGAGCGAGGTATGTAGGCGGTGCTACAGAGT 585
CGGTGACCGTCGTCGGTGACCATTGTCCTAATCGTCTCGCTCCATACATCCGCCACGATGTCTCA
ori

TCTTGAAGTGGTGGCCTAACTACGGCTACACTAGAAGGACAGTATTTGGTATCTGCGCTCTGCTG 650
AGAACTTCACCACCGGATTGATGCCGATGTGATCTTCCTGTCATAAACCATAGACGCGAGACGAC
ori

AAGCCAGTTACCTTCGGAAAAAGAGTTGGTAGCTCTTGATCCGGCAAACAAACCACCGCTGGTAG 715
TTCGGTCAATGGAAGCCTTTTTCTCAACCATCGAGAACTAGGCCGTTTGTTTGGTGGCGACCATC
ori

CGGTGGTTTTTTTGTTTGCAAGCAGCAGATTACGCGCAGAAAAAAAGGATCTCAAGAAGATCCTT 780
GCCACCAAAAAAACAAACGTTCGTCGTCTAATGCGCGTCTTTTTTTCCTAGAGTTCTTCTAGGAA
ori

TGATCTTTTCTACGGGGTCTGACGCTCAGTGGAACGAAAACTCACGTTAAGGGATTTTGGTCATG 845
ACTAGAAAAGATGCCCCAGACTGCGAGTCACCTTGCTTTTGAGTGCAATTCCCTAAAACCAGTAC

```
AGATTATCAAAAAGGATCTTCACCTAGATCCTTTTAAATTAAAAATGAAGTTTTAAATCAATCTA
                                                                   910
TCTAATAGTTTTTCCTAGAAGTGGATCTAGGAAAATTTAATTTTTACTTCAAAATTTAGTTAGAT

AAGTATATATGAGTAAACTTGGTCTGACAGTTACCAATGCTTAATCAGTGAGGCACCTATCTCAG
                                                                   975
TTCATATATACTCATTTGAACCAGACTGTCAATGGTTACGAATTAGTCACTCCGTGGATAGAGTC
                    AmpR

CGATCTGTCTATTTCGTTCATCCATAGTTGCCTGACTCCCCGTCGTGTAGATAACTACGATACGG
                                                                   1040
GCTAGACAGATAAAGCAAGTAGGTATCAACGGACTGAGGGGCAGCACATCTATTGATGCTATGCC
                    AmpR

GAGGGCTTACCATCTGGCCCCAGTGCTGCAATGATACCGCGAGACCCACGCTCACCGGCTCCAGA
                                                                   1105
CTCCCGAATGGTAGACCGGGGTCACGACGTTACTATGGCGCTCTGGGTGCGAGTGGCCGAGGTCT
                    AmpR

TTTATCAGCAATAAACCAGCCAGCCGGAAGGGCCGAGCGCAGAAGTGGTCCTGCAACTTTATCCG
                                                                   1170
AAATAGTCGTTATTTGGTCGGTCGGCCTTCCCGGCTCGCGTCTTCACCAGGACGTTGAAATAGGC
                    AmpR

CCTCCATCCAGTCTATTAATTGTTGCCGGGAAGCTAGAGTAAGTAGTTCGCCAGTTAATAGTTTG
                                                                   1235
GGAGGTAGGTCAGATAATTAACAACGGCCCTTCGATCTCATTCATCAAGCGGTCAATTATCAAAC
                    AmpR

CGCAACGTTGTTGCCATTGCTACAGGCATCGTGGTGTCACGCTCGTCGTTTGGTATGGCTTCATT
                                                                   1300
GCGTTGCAACAACGGTAACGATGTCCGTAGCACCACAGTGCGAGCAGCAAACCATACCGAAGTAA
                    AmpR

CAGCTCCGGTTCCCAACGATCAAGGCGAGTTACATGATCCCCCATGTTGTGCAAAAAAGCGGTTA
                                                                   1365
GTCGAGGCCAAGGGTTGCTAGTTCCGCTCAATGTACTAGGGGGTACAACACGTTTTTTCGCCAAT
                    AmpR

GCTCCTTCGGTCCTCCGATCGTTGTCAGAAGTAAGTTGGCCGCAGTGTTATCACTCATGGTTATG
                                                                   1430
CGAGGAAGCCAGGAGGCTAGCAACAGTCTTCATTCAACCGGCGTCACAATAGTGAGTACCAATAC
                    AmpR

GCAGCACTGCATAATTCTCTTACTGTCATGCCATCCGTAAGATGCTTTTCTGTGACTGGTGAGTA
                                                                   1495
CGTCGTGACGTATTAAGAGAATGACAGTACGGTAGGCATTCTACGAAAAGACACTGACCACTCAT
                    AmpR

tTCAACCAAGTCATTCTGAGAATAGTGTATGCGGCGACCGAGTTGCTCTTGCCCGGCGTCAATAC
                                                                   1560
aAGTTGGTTCAGTAAGACTCTTATCACATACGCCGCTGGCTCAACGAGAACGGGCCGCAGTTATG
                    AmpR

GGGATAATACCGCGCCACATAGCAGAACTTTAAAAGTGCTCATCATTGGAAAACGTTCTTCGGGG
                                                                   1625
CCCTATTATGGCGCGGTGTATCGTCTTGAAATTTTCACGAGTAGTAACCTTTTGCAAGAAGCCCC
                    AmpR

CGAAAACTCTCAAGGATCTTACCGCTGTTGAGATCCAGTTCGATGTAACCCACTCGTGCACCCAA
                                                                   1690
GCTTTTGAGAGTTCCTAGAATGGCGACAACTCTAGGTCAAGCTACATTGGGTGAGCACGTGGGTT
                    AmpR
```

CTGATCTTCAGCATCTTTTACTTTCACCAGCGTTTCTGGGTGAGCAAAAACAGGAAGGCAAAATG 1755
GACTAGAAGTCGTAGAAAATGAAAGTGGTCGCAAAGACCCACTCGTTTTTGTCCTTCCGTTTTAC
AmpR

CCGCAAAAAAGGGAATAAGGGCGACACGGAAATGTTGAATACTCATACTCTTCCTTTTTCAATAT 1820
GGCGTTTTTTCCCTTATTCCCGCTGTGCCTTTACAACTTATGAGTATGAGAAGGAAAAAGTTATA
AmpR | AmpR promoter

TATTGAAGCATTTATCAGGGTTATTGTCTCATGAGCGGATACATATTTGAATGTATTTAGAAAAA 1885
ATAACTTCGTAAATAGTCCCAATAACAGAGTACTCGCCTATGTATAAACTTACATAAATCTTTTT
AmpR promoter

TAAACAAATAGGGGTTCCGCGCACATTTCCCCGAAAAGTGCCACCTGACGTCTAAGAAACCATTA 1950
ATTTGTTTATCCCCAAGGCGCGTGTAAAGGGGCTTTTCACGGTGGACTGCAGATTCTTTGGTAAT
AmpR promoter

TTATCATGACATTAACCTATAAAAATAGGCGTATCACGAGGCCCTTTCGTCTCGCGCGTTTCGGT 2015
AATAGTACTGTAATTGGATATTTTTATCCGCATAGTGCTCCGGGAAAGCAGAGCGCGCAAAGCCA

GATGACGGTGAAAACCTCTGACACATGCAGCTCCCGGAGACGGTCACAGCTTGTCTGTAAGCGGA 2080
CTACTGCCACTTTTGGAGACTGTGTACGTCGAGGGCCTCTGCCAGTGTCGAACAGACATTCGCCT

TGCCGGGAGCAGACAAGCCCGTCAGGGCGCGTCAGCGGGTGTTGGCGGGTGTCGGGGCTGGCTTA 2145
ACGGCCCTCGTCTGTTCGGGCAGTCCCGCGCAGTCGCCCACAACCGCCCACAGCCCCGACCGAAT

TatI
ACTATGCGGCATCAGAGCAGATTGTACTGAGAGTGCACCATAAAATTGTAAACGTTAATATTTTG 2210
TGATACGCCGTAGTCTCGTCTAACATGACTCTCACGTGGTATTTTAACATTTGCAATTATAAAAC
m13 ori

TTAAAATTCGCGTTAAATTTTTGTTAAATCAGCTCATTTTTTAACCAATAGGCCGAAATCGGCAA 2275
AATTTTAAGCGCAATTTAAAAACAATTTAGTCGAGTAAAAAATTGGTTATCCGGCTTTAGCCGTT
m13 ori

PsiI
AATCCCTTATAAATCAAAAGAATAGCCCGAGATAGGGTTGAGTGTTGTTCCAGTTTGGAACAAGA 2340
TTAGGGAATATTTAGTTTTCTTATCGGGCTCTATCCCAACTCACAACAAGGTCAAACCTTGTTCT
m13 ori

GTCCACTATTAAAGAACGTGGACTCCAACGTCAAAGGGCGAAAAACCGTCTATCAGGGCGATGGC 2405
CAGGTGATAATTTCTTGCACCTGAGGTTGCAGTTTCCCGCTTTTTGGCAGATAGTCCCGCTACCG
m13 ori

CCACTACGTGAACCATCACCCAAATCAAGTTTTTTGGGGTCGAGGTGCCGTAAAGCACTAAATCG 2470
GGTGATGCACTTGGTAGTGGGTTTAGTTCAAAAAACCCCAGCTCCACGGCATTTCGTGATTTAGC
m13 ori

GAACCCTAAAGGGAGCCCCCGATTTAGAGCTTGACGGGGAAAGCCGGCGAACGTGGCGAGAAAGG 2535
CTTGGGATTTCCCTCGGGGGCTAAATCTCGAACTGCCCCTTTCGGCCGCTTGCACCGCTCTTTCC
m13 ori

AAGGGAAGAAAGCGAAAGGAGCGGGCGCTAGGGCGCTGGCAAGTGTAGCGGTCACGCTGCGCGTA 2600
TTCCCTTCTTTCGCTTTCCTCGCCCGCGATCCCGCGACCGTTCACATCGCCAGTGCGACGCGCAT
m13 ori

ACCACCACACCCGCCGCGCTTAATGCGCCGCTACAGGGCGCGTACTATGGTTGCTTTGACGTATG 2665
TGGTGGTGTGGGCGGCGCGAATTACGCGGCGATGTCCCGCGCATGATACCAACGAAACTGCATAC
m13 ori

KasI NarI SfoI PluTI
CGGTGTGAAATACCGCACAGATGCGTAAGGAGAAAATACCGCATCAGGCGCCATTCGCCATTCAG 2730
GCCACACTTTATGGCGTGTCTACGCATTCCTCTTTTATGGCGTAGTCCGCGGTAAGCGGTAAGTC

GCTGCGCAACTGTTGGGAAGGGCGATCGGTGCGGGCCTCTTCGCTATTACGCCAGCTGGCGAAAG 2795
CGACGCGTTGACAACCCTTCCCGCTAGCCACGCCCGGAGAAGCGATAATGCGGTCGACCGCTTTC

GGGGATGTGCTGCAAGGCGATTAAGTTGGGTAACGCCAGGGTTTTCCCAGTCACGACGTTGTAAA 2860
CCCCTACACGACGTTCCGCTAATTCAACCCATTGCGGTCCCAAAAGGGTCAGTGCTGCAACATTT
m13 fwd

ACGACGGCCAGTGAATTAATTCACCGCCGTATGGCTGACCGGCGATTACTAGCGATTCCGGCTTC 2925
TGCTGCCGGTCACTTAATTAAGTGGCGGCATACCGACTGGCCGCTAATGATCGCTAAGGCCGAAG
m13 fwd
pPRV1-II targeting
16S rRNA
EcoRI end of targeting 104,085

ATGCAGGCGAGTTGCAGCCTGCAATCCGAACTGAGGACGGGTTTTTGGGGTTAGCTCACCCTCGC 2990
TACGTCCGCTCAACGTCGGACGTTAGGCTTGACTCCTGCCCAAAAACCCCAATCGAGTGGGAGCG
pPRV1-II targeting
16S rRNA

NruI* PmlI
GGGATCGCGACCCTTTGTCCCGGCCATTGTAGCACGTGTGTCGCCCAGGGCATAAGGGGCATGAT 3055
CCCTAGCGCTGGGAAACAGGGCCGGTAACATCGTGCACACAGCGGGTCCCGTATTCCCCGTACTA
pPRV1-II targeting
16S rRNA

GACTTGACGTCATCCTCACCTTCCTCCGGCTTATCACCGGCAGTCTGTTCAGGGTTCCAAACTCA 3120
CTGAACTGCAGTAGGAGTGGAAGGAGGCCGAATAGTGGCCGTCAGACAAGTCCCAAGGTTTGAGT
pPRV1-II targeting
16S rRNA

ACGATGGCAACTAAACACGAGGGTTGCGCTCGTTGCGGGACTTAACCCAACACCTTACGGCACGA 3185
TGCTACCGTTGATTTGTGCTCCCAACGCGAGCAACGCCCTGAATTGGGTTGTGGAATGCCGTGCT
pPRV1-II targeting
16S rRNA

GCTGACGACAGCCATGCACCACCTGTGTCCGCGTTCCCGAAGGCACCCCTCTCTTTCAAGAGGAT 3250
CGACTGCTGTCGGTACGTGGTGGACACAGGCGCAAGGGCTTCCGTGGGGAGAGAAAGTTCTCCTA
pPRV1-II targeting
16S rRNA

TCGCGGCATGTCAAGCCCTGGTAAGGTTCTTCGCTTTGCATCGAATTAAACCACATGCTCCACCG 3315
AGCGCCGTACAGTTCGGGACCATTCCAAGAAGCGAAACGTAGCTTAATTTGGTGTACGAGGTGGC
pPRV1-II targeting
16S rRNA

PspOMI ApaI
CTTGTGCGGGCCCCCGTCAATTCCTTTGAGTTTCATTCTTGCGAACGTACTCCCCAGGCGGGATA 3380
GAACACGCCCGGGGGCAGTTAAGGAAACTCAAAGTAAGAACGCTTGCATGAGGGGTCCGCCCTAT
pPRV1-II targeting
16S rRNA

BsgI
CTTAACGCGTTAGCTACAGCACTGCACGGGTCGATACGCACAGCGCCTAGTATCCATCGTTTACG 3445
GAATTGCGCAATCGATGTCGTGACGTGCCCAGCTATGCGTGTCGCGGATCATAGGTAGCAAATGC
pPRV1-II targeting
16S rRNA

GCTAGGACTACTGGGGTATCTAATCCCATTCGCTCCCCTAGCTTTCGTCTCTCAGTGTCAGTGTC 3510
CGATCCTGATGACCCCATAGATTAGGGTAAGCGAGGGGATCGAAAGCAGAGAGTCACAGTCACAG
pPRV1-II targeting
16S rRNA

GGCCCAGCAGAGTGCTTTCGCCGTTGGTGTTCTTTCCGATCTCTACGCATTTCACCGCTCCACCG 3575
CCGGGTCGTCTCACGAAAGCGGCAACCACAAGAAAGGCTAGAGATGCGTAAAGTGGCGAGGTGGC
pPRV1-II targeting
16S rRNA

GAAATTCCCTCTGCCCCTACCGTACTCCAGCTTGGTAGTTTCCACCGCCTGTCCAGGGTTGAGCC 3640
CTTTAAGGGAGACGGGGATGGCATGAGGTCGAACCATCAAAGGTGGCGGACAGGTCCCAACTCGG
pPRV1-II targeting
16S rRNA

BspEI
CTGGGATTTGACGGCGGACTTAAAAAGCCACCTACAGACGCTTTACGCCCAATCATTCCGGATAA 3705
GACCCTAAACTGCCGCCTGAATTTTTCGGTGGATGTCTGCGAAATGCGGGTTAGTAAGGCCTATT
pPRV1-II targeting
16S rRNA

CGCTTGCATCCTCTGTATTACCGCGGCTGCTGGCACAGAGTTAGCCGATGCTTATTCCCCAGATA 3770
GCGAACGTAGGAGACATAATGGCGCCGACGACCGTGTCTCAATCGGCTACGAATAAGGGGTCTAT
pPRV1-II targeting
16S rRNA

C C G T C A T T G C T T C T T C T C C G G G A A A A G A A G T T C A C G A C C C G T G G G C C T T C T A C C T C C A C G C G G C A
3835
G G C A G T A A C G A A G A A G A G G C C C T T T T C T T C A A G T G C T G G G C A C C C G G A A G A T G G A G G T G C G C C G T
pPRV1-II targeting
16S rRNA

T T G C T C C G T C A G G C T T T C G C C C A T T G C G G A A A A T T C C C C A C T G C T G C C T C C C G T A G G A G T C T G G G
3900
A A C G A G G C A G T C C G A A A G C G G G T A A C G C C T T T T A A G G G G T G A C G A C G G A G G G C A T C C T C A G A C C C
pPRV1-II targeting
16S rRNA

C C G T G T C T C A G T C C C A G T G T G G C T G A T C A T C C T C T C G G A C C A G C T A C T G A T C A T C G C C T T G G T A A
3965
G G C A C A G A G T C A G G G T C A C A C C G A C T A G T A G G A G A G C C T G G T C G A T G A C T A G T A G C G G A A C C A T T
pPRV1-II targeting
16S rRNA

G C T A T T G C C T C A C C A A C T A G C T A A T C A G A C G C G A G C C C C T C C T C G G G C G G A T T C C T C C T T T T G C T
4030
C G A T A A C G G A G T G G T T G A T C G A T T A G T C T G C G C T C G G G G A G G A G C C C G C C T A A G G A G G A A A A C G A
pPRV1-II targeting
16S rRNA

BbvCI
C C T C A G C C T A C G G G G T A T T A G C A G C C G T T T C C A G C T G T T G T T C C C C T C C C A A G G G C A G G T T C T T A
4095
G G A G T C G G A T G C C C C A T A A T C G T C G G C A A A G G T C G A C A A C A A G G G G A G G G T T C C C G T C C A A G A A T
pPRV1-II targeting
16S rRNA

C G C G T T A C T C A C C C G T C C G C C A C T G G A A A C A C C A C T T C C C G T C C G A C T T G C A T G T G T T A A G C A T G
4160
G C G C A A T G A G T G G G C A G G C G G T G A C C T T T G T G G T G A A G G G C A G G C T G A A C G T A C A C A A T T C G T A C
pPRV1-II targeting
16S rRNA

C C G C C A G C G T T C A T C C T G A G C C A G G A T C G A A C T C T C C A T G A G A T T C A T A G T T G C A T T A C T T A T A G
4225
G G C G G T C G C A A G T A G G A C T C G G T C C T A G C T T G A G A G G T A C T C T A A G T A T C A A C G T A A T G A A T A T C
pPRV1-II targeting
16S rRNA

C T T C C T T G T T C G T A G A C A A A G C G G A T T C G G A A T T G T C T T T C A T T C C A A G G C A T A A C T T G T A T C C A
4290
G A A G G A A C A A G C A T C T G T T T C G C C T A A G C C T T A A C A G A A A G T A A G G T T C C G T A T T G A A C A T A G G T
pPRV1-II targeting

T G C G C T T C A T A T T C G C C C G G A G T T C G C T C C C A G A A A T A T A G C C A T C C C T G C C C C C T C A C G T C A A T
4355
A C G C G A A G T A T A A G C G G G C C T C A A G C G A G G G T C T T T A T A T C G G T A G G G A C G G G G G A G T G C A G T T A
pPRV1-II targeting

StuI
C C C A C G A G C C T C T T A T C C A T T C T C A T T G A A C G A C G G C G G G G G A G C T T T C G A G G C C T C G A A A T C C A
4420
G G G T G C T C G G A G A A T A G G T A A G A G T A A C T T G C T G C C G C C C C C T C G A A A G C T C C G G A G C T T T A G G T
pPRV1-II targeting

ACTAGAAAAACTCACATTGGGCTTAGGGATAATCAGGCTCGAACTGATGACTTCCACCACGTCAA 4485
TGATCTTTTTGAGTGTAACCCGAATCCCTATTAGTCCGAGCTTGACTACTGAAGGTGGTGCAGTT

pPRV1-II targeting

trnV

GGTGACACTCTACCGCTGAGTTATATCCCTTCCCCGCCCCATCGAGAAATAGAACTGACTAATCC 4550
CCACTGTGAGATGGCGACTCAATATAGGGAAGGGGCGGGGTAGCTCTTTATCTTGACTGATTAGG

pPRV1-II targeting

trnV

TAAGTCAAAGGGTCGAGAAACTCAACGCCACTATTCTTGAACAACTTGGAGCCGGGCCTTCTTTT 4615
ATTCAGTTTCCCAGCTCTTTGAGTTGCGGTGATAAGAACTTGTTGAACCTCGGCCCGGAAGAAAA

pPRV1-II targeting

MfeI PacI

CGCACTATTACGGATATGAAAATAATGGTCAAAATCGGATTCAATTGTCAAAGTCCTTTAATTAA 4680
GCGTGATAATGCCTATACTTTTATTACCAGTTTTAGCCTAAGTTAACAGTTTCAGGAAATTAATT

pPRV1-II targeting

EcoRI Eco53kI SacI Acc65I KpnI XbaI SalI PstI SbfI HindIII

GAATTCGAGCTCGGTACCCGGGGATCCTCTAGAGTCGACCTGCAGGCATGCAAGCTTAAGTCTTT 4745
CTTAAGCTCGAGCCATGGGCCCCTAGGAGATCTCAGCTGGACGTCCGTACGTTCGAATTCAGAAA

MCS

Medicago ptDNA 60118-60264

ACAATGACAATGGAAACCGATGTAAAGGGATGTAGCGCAGCTTGGTAGCGCGTTTGTTTTGGGTA 4810
TGTTACTGTTACCTTTGGCTACATTTCCCTACATCGCGTCGAACCATCGCGCAAACAAAACCCAT

Medicago ptDNA 60118-60264

trnP coding

CAAAATGTCACAGGTTCAAATCCTGTCATCCCTATCCCTAACTTGTAGTTATCGTATCAGCAGTA 4875
GTTTTACAGTGTCCAAGTTTAGGACAGTAGGGATAGGGATTGAACATCAATAGCATAGTCGTCAT

Medicago ptDNA 60118-60264

trnP coding

ACAATAGATGTGCCAGGGCGTGCCCTTGGGCTCCCCGGGCGCGTTGGGTTGCGCTATATATATGA 4940
TGTTATCTACACGGTCCCGCACGGGAACCCGAGGGGCCCGCGCAACCCAACGCGATATATATACT

attB PrbcL

Medicago ptDNA 60118-60264

AAGAGTATACAATAATGATGTATTTGAGTTGTAGGGAGGGATTTATGGGGGAAGCGGTGATCGCC 5005
TTCTCATATGTTATTACTACATAAACTCAACATCCCTCCCTAAATACCCCCTTCGCCACTAGCGG

1 5
Met Gly Glu Ala Val Ile Ala

PrbcL rbcL 5'UTR SmR

GAAGTATCGACTCAACTATCAGAGGTAGTTGGCGTCATCGAGCGCCATCTCGAACCGACGTTGCT 5070
CTTCATAGCTGAGTTGATAGTCTCCATCAACCGCAGTAGCTCGCGGTAGAGCTTGGCTGCAACGA

10 15 20 25
Glu Val Ser Thr Gln Leu Ser Glu Val Val Gly Val Ile Glu Arg His Leu Glu Pro Thr Leu Leu

SmR

GGCCGTACATTTGTACGGCTCCGCAGTGGATGGCGGCCTGAAGCCACACAGTGATATTGATTTGC 5135
CCGGCATGTAAACATGCCGAGGCGTCACCTACCGCCGGACTTCGGTGTGTCACTATAACTAAACG
30 35 40 45 50
Ala Val His Leu Tyr Gly Ser Ala Val Asp Gly Gly Leu Lys Pro His Ser Asp Ile Asp Leu
SmR

TGGTTACGGTGACCGTAAGGCTTGATGAAACAACGCGGCGAGCTTTGATCAACGACCTTTTGGAA 5200
ACCAATGCCACTGGCATTCCGAACTACTTTGTTGCGCCGCTCGAAACTAGTTGCTGGAAAACCTT
55 60 65 70
Leu Val Thr Val Thr Val Arg Leu Asp Glu Thr Thr Arg Arg Ala Leu Ile Asn Asp Leu Leu Glu
SmR

ACTTCGGCTTCCCCTGGAGAGAGCGAGATTCTCCGCGCTGTAGAAGTCACCATTGTTGTGCACGA 5265
TGAAGCCGAAGGGGACCTCTCTCGCTCTAAGAGGCGCGACATCTTCAGTGGTAACAACACGTGCT
75 80 85 90
Thr Ser Ala Ser Pro Gly Glu Ser Glu Ile Leu Arg Ala Val Glu Val Thr Ile Val Val His Asp
SmR

BlpI

CGACATCATTCCGTGGCGTTATCCAGCTAAGCGCGAACTGCAATTTGGAGAATGGCAGCGCAATG 5330
GCTGTAGTAAGGCACCGCAATAGGTCGATTCGCGCTTGACGTTAAACCTCTTACCGTCGCGTTAC
95 100 105 110 115
Asp Ile Ile Pro Trp Arg Tyr Pro Ala Lys Arg Glu Leu Gln Phe Gly Glu Trp Gln Arg Asn
SmR

ACATTCTTGCAGGTATCTTCGAGCCAGCCACGATCGACATTGATCTGGCTATCTTGCTGACAAAA 5395
TGTAAGAACGTCCATAGAAGCTCGGTCGGTGCTAGCTGTAACTAGACCGATAGAACGACTGTTTT
120 125 130 135
Asp Ile Leu Ala Gly Ile Phe Glu Pro Ala Thr Ile Asp Ile Asp Leu Ala Ile Leu Leu Thr Lys
SmR

GCAAGAGAACATAGCGTTGCCTTGGTAGGTCCAGCGGCGGAGGAACTCTTTGATCCGGTTCCTGA 5460
CGTTCTCTTGTATCGCAACGGAACCATCCAGGTCGCCGCCTCCTTGAGAAACTAGGCCAAGGACT
140 145 150 155
Ala Arg Glu His Ser Val Ala Leu Val Gly Pro Ala Ala Glu Glu Leu Phe Asp Pro Val Pro Glu
SmR

ACAGGATCTATTTGAGGCGCTAAATGAAACCTTAACGCTATGGAACTCGCCGCCCGACTGGGCTG 5525
TGTCCTAGATAAACTCCGCGATTTACTTTGGAATTGCGATACCTTGAGCGGCGGGCTGACCCGAC
160 165 170 175 180
Gln Asp Leu Phe Glu Ala Leu Asn Glu Thr Leu Thr Leu Trp Asn Ser Pro Pro Asp Trp Ala
SmR

GCGATGAGCGAAATGTAGTGCTTACGTTGTCCCGCATTTGGTACAGCGCAGTAACCGGCAAAATC 5590
CGCTACTCGCTTTACATCACGAATGCAACAGGGCGTAAACCATGTCGCGTCATTGGCCGTTTTAG
185 190 195 200
Gly Asp Glu Arg Asn Val Val Leu Thr Leu Ser Arg Ile Trp Tyr Ser Ala Val Thr Gly Lys Ile
SmR

GCGCCGAAGGATGTCGCTGCCGACTGGGCAATGGAGCGCCTGCCGGCCCAGTATCAGCCCGTCAT 5655
CGCGGCTTCCTACAGCGACGGCTGACCCGTTACCTCGCGGACGGCCGGGTCATAGTCGGGCAGTA
205 210 215 220
Ala Pro Lys Asp Val Ala Ala Asp Trp Ala Met Glu Arg Leu Pro Ala Gln Tyr Gln Pro Val Ile
SmR

BssHII

ACTTGAAGCTAGACAGGCTTATCTTGGACAAGAAGAAGATCGCTTGGCCTCGCGCGCAGATCAGT 5720
TGAACTTCGATCTGTCCGAATAGAACCTGTTCTTCTTCTAGCGAACCGGAGCGCGCGTCTAGTCA

225 Leu Glu Ala Arg Gln 230 Ala Tyr Leu Gly Gln 235 Glu Glu Asp Arg Leu 240 Ala Ser Arg Ala Asp 245 Gln
SmR

BstXI

TGGAAGAATTTGTCCACTACGTGAAAGGCGAGATCACCAAGGTAGTGGGCAAAGAACAAAAACTC 5785
ACCTTCTTAAACAGGTGATGCACTTTCCGCTCTAGTGGTTCCATCACCCGTTTCTTGTTTTTGAG

Leu Glu Glu Phe 250 Val His Tyr Val Lys 255 Gly Glu Ile Thr Lys 260 Val Val Gly Lys | 1 Glu Gln Lys Leu
SmR | Myc

BbsI

ATTTCTGAAGAAGACTTGTGAGATCCTGGCCTAGTCTATAGGAGGTTTTGAAAAGAAAGGAGCAA 5850
TAAAGACTTCTTCTGAACACTCTAGGACCGGATCAGATATCCTCCAAAACTTTTCTTTCCTCGTT

5 Ile Ser Glu Glu Asp 10 Leu *
Myc | TpsbA 3'UTR

TAATCATTTTCTTGTTCTATCAAGAGGGTGCTATTGCTCCTTTCTTTTTTTCTTTTTATTTATTT 5915
ATTAGTAAAAGAACAAGATAGTTCTCCCACGATAACGAGGAAAGAAAAAAAGAAAAATAAATAAA

TpsbA 3'UTR

SpeI

ACTAGTATTTTACTTACATAGACTTTTTTGTTTACATTATAGAAAAAGAAGGAGAGGTTATTTTC 5980
TGATCATAAAATGAATGTATCTGAAAAAACAAATGTAATATCTTTTTCTTCCTCTCCAATAAAAG

TpsbA 3'UTR

NotI
EagI

TTGCATTTATTCATGCCCCAACTGGGGTAACCTTTGAGTTCTCTCAGTTGGGGGGCGGCCGCAGG 6045
AACGTAAATAAGTACGGGGTTGACCCCATTGGAAACTCAAGAGAGTCAACCCCCCGCCGGCGTCC

TpsbA 3'UTR | attP

ACTTTAACTGCCCCTATCGGAAATAGGATTGACTACCGATTCCGAAGGAACTGGAGTTACATCTC 6110
TGAAATTGACGGGGATAGCCTTTATCCTAACTGATGGCTAAGGCTTCCTTGACCTCAATGTAGAG

pPRV1-II targeting

TTTTCCATTCAAGAGTTCTTATGCGTTTCCACGCCCCTTTGAGACCCCGAAAAATGGACAAATTC 6175
AAAAGGTAAGTTCTCAAGAATACGCAAAGGTGCGGGGAAACTCTGGGGCTTTTTACCTGTTTAAG

pPRV1-II targeting

CTTTTCTTAGGAACACATACAAGATTCGTCACTACAAAAAGGATAATGGTAACCCTACCATTAAC 6240
GAAAAGAATCCTTGTGTATGTTCTAAGCAGTGATGTTTTTCCTATTACCATTGGGATGGTAATTG

pPRV1-II targeting

TACTTCATTTATGAATTTCATAGTAATAGAAATACATGTCCTACCGAGACAGAATTTGGAACTTG 6305
ATGAAGTAAATACTTAAAGTATCATTATCTTTATGTACAGGATGGCTCTGTCTTAAACCTTGAAC

pPRV1-II targeting

rps12/7 TSS

C T A T C C T C T T G C C T A G C A G G C A A A G A T T T A C C T C C G T G G A A A G G A T G A T T C A T T C G G A T C G A C A T
6370
G A T A G G A G A A C G G A T C G T C C G T T T C T A A A T G G A G G C A C C T T T C C T A C T A A G T A A G C C T A G C T G T A
pPRV1-II targeting

G A G A G T C C A A C T A C A T T G C C A G A A T C C A T G T T G T A T A T T T G A A A G A G G T T G A C C T C C T T G C T T C T
6435
C T C T C A G G T T G A T G T A A C G G T C T T A G G T A C A A C A T A T A A A C T T T C T C C A A C T G G A G G A A C G A A G A
pPRV1-II targeting

C T C A T G G T A C A C T C C T C T T C C C G C C G A G C C C C T T T T C T C C T C G G T C C A C A G A G A C A A A A T G T A G G
6500
G A G T A C C A T G T G A G G A G A A G G G C G G C T C G G G G A A A A G A G G A G C C A G G T G T C T C T G T T T T A C A T C C
pPRV1-II targeting

A C T G G T G C C A A C A A T T C A T C A G A C T C A C T A A G T C G G G A T C A C T A A C T A A T A C T A A T C T A A T A T A A
6565
T G A C C A C G G T T G T T A A G T A G T C T G A G T G A T T C A G C C C T A G T G A T T G A T T A T G A T T A G A T T A T A T T
pPRV1-II targeting

T A G T C T A A T A T A T C T A A T A T A A T A G A A A A T A C T A A T A T A A T A G A A A A G A A C T G T C T T T T C T G T A T
6630
A T C A G A T T A T A T A G A T T A T A T T A T C T T T T A T G A T T A T A T T A T C T T T T C T T G A C A G A A A A G A C A T A
pPRV1-II targeting

EcoRV
A C T T T C C C C G G T T C C G T T G C T A C C G C G G G C T T T A C G C A A T C G A T C G G A T T A G A T A G A T A T C C C T T
6695
T G A A A G G G G C C A A G G C A A C G A T G G C G C C C G A A A T G C G T T A G C T A G C C T A A T C T A T C T A T A G G G A A
pPRV1-II targeting

C A A C A T A G G T C A T C G A A A G G A T C T C G G A G A C C C A C C A A A G T A C G A A A G C C A G G A T C T T T C A G A A A
6760
G T T G T A T C C A G T A G C T T T C C T A G A G C C T C T G G G T G G T T T C A T G C T T T C G G T C C T A G A A A G T C T T T
pPRV1-II targeting

A C G G A T T C C T A T T C A A A G A G T G C A T A A C C G C A T G G A T A A G C T C A C A C T A A C C C G T C A A T T T G G G A
6825
T G C C T A A G G A T A A G T T T C T C A C G T A T T G G C G T A C C T A T T C G A G T G T G A T T G G G C A G T T A A A C C C T
pPRV1-II targeting

T C C A A A T T C G A G A T T T T C C T T G G G A G G T A T C G G G A A G G A T T T G G A A T G G A A T A A T A T C G A T T C A T
6890
A G G T T T A A G C T C T A A A A G G A A C C C T C C A T A G C C C T T C C T A A A C C T T A C C T T A T T A T A G C T A A G T A
pPRV1-II targeting

A C A G A A G A A A A G G T T C T C T A T T G A T T C A A A C A C T G T A C C T A A C C T A T G G G A T A G G G A T C G A G G A A
6955
T G T C T T C T T T T C C A A G A G A T A A C T A A G T T T G T G A C A T G G A T T G G A T A C C C T A T C C C T A G C T C C T T
pPRV1-II targeting

G G G G A A A A A C C G A A G A T T T C A C A T G G T A C T T T T A T C A A T C T G A T T T A T T T C G T A C C T T T C G T T C A
7020
C C C C T T T T T G G C T T C T A A A G T G T A C C A T G A A A A T A G T T A G A C T A A A T A A A G C A T G G A A A G C A A G T
pPRV1-II targeting

A T G A G A A A A T G G G T C A A A T T C T A C A G G A T C A A A C C T A T G G G A C T T A A G G A A T G A T A T A A A A A A A A
7085
T A C T C T T T T A C C C A G T T T A A G A T G T C C T A G T T T G G A T A C C C T G A A T T C C T T A C T A T A T T T T T T T T
pPRV1-II targeting

GAGAGGGAAAATATTCATATTAAATAAATATGAAGTAGAAGAACCCAGATTCCAAATGAACAAAT 7150
CTCTCCCTTTTATAAGTATAATTTATTTATACTTCATCTTCTTGGGTCTAAGGTTTACTTGTTTA

pPRV1-II targeting

TCAAACTTGAAAAGGATCTTCCTTATTCTTGAAGAATGAGGGGCAAAGGGATTGATCAAGAAAGA 7215
AGTTTGAACTTTTCCTAGAAGGAATAAGAACTTCTTACTCCCCGTTTCCCTAACTAGTTCTTTCT

pPRV1-II targeting

BglII end of ptDNA 101,146

TCCTCTAGCTAGAGCTTGGCGTAATCATGGTCATAGCTGTTTCCTGTGTGAAATTGTTATCCGCT 7280
AGGAGATCGATCTCGAACCGCATTAGTACCAGTATCGACAAAGGACACACTTTAACAATAGGCGA

pPRV1-II targeting | m13 rev primer | lac operator

BglII end of ptDNA 101,146

CACAATTCCACACAACATACGAGCCGGAAGCATAAAGTGTAAAGCCTGGGGTGCCTAATGAGTGA 7345
GTGTTAAGGTGTGTTGTATGCTCGGCCTTCGTATTTCACATTTCGGACCCCACGGATTACTCACT

lac operator | Lac promoter

GCTAACTCACATTAATTGCGTTGCGCTCACTGCCCGCTTTCCAGTCGGGAAACCTGTCGTGCCAG 7410
CGATTGAGTGTAATTAACGCAACGCGAGTGACGGGCGAAAGGTCAGCCCTTTGGACAGCACGGTC

CTGCATTAATGAATCGGCCAACGCGCGGGGAGAGGCGGTTTGCGTATTGGGCGCT ••• 3′ 7465
GACGTAATTACTTAGCCGGTTGCGCGCCCCTCTCCGCCAAACGCATAACCCGCGA 5′

	Feature	Location	Size	Direction	Type
✓	**ori**	182 .. 770	589 bp	⊢⊣	misc_feature
✓	**AmpR**	941 .. 1801	861 bp	←	misc_feature
	/note = Ampicilin r				
✓	**AmpR promoter**	1802 .. 1906	105 bp	⊢⊣	misc_feature
✓	**m13 ori**	2188 .. 2643	456 bp	⊢⊣	misc_feature
✓	**m13 fwd**	2856 .. 2872	17 bp	⊢⊣	misc_feature
✓	**pPRV1-II targeting**	2878 .. 4669	1792 bp	⊢⊣	misc_feature
✓	**16S rRNA**	2878 .. 4203	1326 bp	←	misc_feature
✓	**EcoRI end of targeting 104,085**	2878 .. 2889	12 bp	⊢⊣	misc_feature
✓	**trnV**	4444 .. 4515	72 bp	←	misc_feature
✓	**MCS**	4681 .. 4737	57 bp	⊢⊣	misc_feature
	/note = pUC18/19 multiple cloning site				
✓	**Medicago ptDNA 60118-60264**	4738 .. 4884	147 bp	⊢⊣	misc_feature
	/note = trnP from *Medicago truncatula* NC_003119.8 between nucleotides 60118-60264				
✓	**trnP coding**	4770 .. 4844	75 bp	→	tRNA
	/note = In GenBank NC_003119.8 - Medicago tuncatula ptDNA; segment is 60118-60264				
✓	**attB**	4885 .. 4918	34 bp	⊢⊣	protein_bind
	/bound_moiety = phage φC31 integrase				
	/note = minimal attB site for the φC31 integrase (Groth et al., 2000)				
✓	**PrbcL**	4919 .. 4966	48 bp	⊢⊣	misc_feature
	/note = tobacco rbcL gene promoter				
✓	**rbcL 5'UTR**	4967 .. 4984	18 bp	⊢⊣	5'UTR
✓	**SmR**	4985 .. 5773	789 bp	→	CDS
	/gene = *aadA*				
	/product = aminoglycoside adenylyltransferase (Murphy, 1985)				
	/note = confers resistance to spectinomycin and streptomycin				
	/translation = MGEAVIAEVSTQLSEVVGVIERHLEPTLLAVHLYGSAVDGGLKPHSDIDLLVTVTVRLDETTRRALINDLLETSASPGESEILRAVEVTIVVHDDIIPWRYPAKRELQFGEWQRND ILAGIFEPATIDIDLAILLTKAREHSVALVGPAAEELFDPVPEQDLFEALNETLTLWNSPPDWAGDERNVVLTLSRIWYSAVTGKIAPKDVAADWAMERLPAQYQPVILEARQAYL GQEEDRLASRADQLEEFVHYVKGEITKVVGK<br>263 amino acids = 29,2 kDa				
✓	**Myc**	5774 .. 5803	30 bp	→	CDS
	/product = Myc (human c-Myc proto-oncogene) epitope tag				
	/translation = EQKLISEEDL<br>10 amino acids = 1,2 kDa				
✓	**TpsbA 3'UTR**	5807 .. 5995	189 bp	⊢⊣	3'UTR
	/note = Tobacco plastid psbA gene 3'UTR				
✓	**attP**	5996 .. 6034	39 bp	⊢⊣	misc_feature
	/note = Integrase attP minimal sequence				
✓	**pPRV1-II targeting**	6046 .. 7217	1172 bp	⊢⊣	misc_feature
✓	**rps12/7 TSS**	6267 .. 6268	2 bp	←	misc_feature
✓	**BglII end of ptDNA 101,146**	7209 .. 7217	9 bp	⊢⊣	misc_feature
✓	**m13 rev primer**	7245 .. 7261	17 bp	⊢⊣	misc_feature
✓	**lac operator**	7269 .. 7285	17 bp	⊢⊣	misc_feature
✓	**Lac promoter**	7293 .. 7323	31 bp	⊢⊣	misc_feature

Sequence: TVV2 eGFP-Fc1cassette copy.dna (Linear / 1835 bp)
Enzymes: New England Biolabs (257 of 678 total)
Features: 9 total

Supplementary Figure 3B

Unique Cutters **Bold**

Start (0)

SacI

5′ GagctcGCTCCCCCGCCGTCGTTCAATGAGAATGGATAAGAGGCTCGTGGGATTGACGTGAGGGG 65
3′ CtcgagCGAGGGGGCGGCAGCAAGTTACTCTTACCTATTCTCCGAGCACCCTAACTGCACTCCCC

Prrn

GCAGGGATGGCTATATTTCTGGGAGTTACTTCTACCCGATAGAGCTTAGAAGTTGGAAGTAATAA 130
CGTCCCTACCGATATAAAGACCCTCAATGAAGATGGGCTATCTCGAATCTTCAACCTTCATTATT

Prrn | Zm atpH 5' UTR

TRS

TTTCTTGGTTGATTGTAtcCTTAACCATTTCTTTTTTTTTGACACGAGGAACTCATCATGGTCTC 195
AAAGAACCAACTAACATagGAATTGGTAAAGAAAAAAAAACTGTGCTCCTTGAGTAGTACCAGAG

1
M V S

Zm atpH 5' UTR | GFP

TAAGGGTGAAGAACTATTTACGGGTGTTGTACCTATTCTCGTCGAACTTGATGGGGATGTCAACG 260
ATTCCCACTTCTTGATAAATGCCCACAACATGGATAAGAGCAGCTTGAACTACCCCTACAGTTGC

5 10 15 20
K G E E L F T G V V P I L V E L D G D V N
GFP

GGCATAAGTTTAGCGTTTCCGGAGAGGGTGAGGGTGATGCTACGTACGGTAAGCTAACGCTTAAA 325
CCGTATTCAAATCGCAAAGGCCTCTCCCACTCCCACTACGATGCATGCCATTCGATTGCGAATTT

25 30 35 40 45
G H K F S V S G E G E G D A T Y G K L T L K
GFP

TTCATCTGCACGACAGGGAAACTGCCCGTTCCTTGGCCGACCCTTGTTACGACGTTGACCTATGG 390
AAGTAGACGTGCTGTCCCTTTGACGGGCAAGGAACCGGCTGGGAACAATGCTGCAACTGGATACC

50 55 60 65
F I C T T G K L P V P W P T L V T T L T Y G
GFP

TGTTCAATGTTTCAGTAGATACCCCGATCAtATGAAGCAACATGATTTTTTTAAGAGTGCAATGC 455
ACAAGTTACAAAGTCATCTATGGGGCTAGTaTACTTCGTTGTACTAAAAAAATTCTCACGTTACG

70 75 80 85
V Q C F S R Y P D H M K Q H D F F K S A M
GFP

CCGAAGGTTACGTTCAAGAGAGGACAATCTTCTTTAAGGACGACGGTAACTACAAAACGCGCGCT 520
GGCTTCCAATGCAAGTTCTCTCCTGTTAGAAGAAATTCCTGCTGCCATTGATGTTTTGCGCGCGA

90 95 100 105 110
P E G Y V Q E R T I F F K D D G N Y K T R A
GFP

GAAGTTAAATTTGAAGGTGATACGTTGGTGAACCGCATCGAGCTTAAGGGTATTGACTTCAAAGA 585
CTTCAATTTAAACTTCCACTATGCAACCACTTGGCGTAGCTCGAATTCCCATAACTGAAGTTTCT

115 120 125 130
E V K F E G D T L V N R I E L K G I D F K E
GFP

GGATGGGAACATCTTAGGTCATAAACTTGAATATAACTATAATTCGCATAACGTATATATTATGG 650
CCTACCCTTGTAGAATCCAGTATTTGAACTTATATTGATATTAAGCGTATTGCATATATAATACC

135 140 145 150
D G N I L G H K L E Y N Y N S H N V Y I M
GFP

```
CTGACAAACAAAAAAACGGTATTAAAGTTAATTTTAAAATTCGACATAACATCGAAGATGGTTCT   715
GACTGTTTGTTTTTTTGCCATAATTTCAATTAAAATTTTAAGCTGTATTGTAGCTTCTACCAAGA
155       160       165       170       175
A D K Q K N G I K V N F K I R H N I E D G S
GFP

GTTCAATTGGCCGATCATTATCAACAAAACACGCCTATCGGGGACGGTCCAGTGCTATTACCCGA   780
CAAGTTAACCGGCTAGTAATAGTTGTTTTGTGCGGATAGCCCCTGCCAGGTCACGATAATGGGCT
180       185       190       195
V Q L A D H Y Q Q N T P I G D G P V L L P D
GFP

CAATCACTATCTTTCAACCCAATCTGCTCTCTCTAAAGACCCCAATGAAAAACGAGACCACATGG   845
GTTAGTGATAGAAAGTTGGGTTAGACGAGAGAGATTTCTGGGGTTACTTTTTGCTCTGGTGTACC
200       205       210       215
N H Y L S T Q S A L S K D P N E K R D H M
GFP

TCTTGCTAGAATTTGTTACtGCaGCGGGTATCACGCTTGGGATGGACGAACTGTATAAAGGGGGG   910
AGAACGATCTTAAACAATGaCGtCGCCCATAGTGCGAACCCTACCTGCTTGACATATTTCCCCCC
220       225       230       235
V L L E F V T A A G I T L G M D E L Y K G G
GFP
3 x G linker

GGTGGTTGCAAGCCCTGTATTTGTACGGTaCCGGAAGTATCTTCTGTGTTCATTTTTCCCCCTAA   975
CCACCAACGTTCGGGACATAAACATGCCAtGGCCTTCATAGAAGACACAAGTAAAAAGGGGGATT
G G C K P C I C T V P E V S S V F I F P P K
(in frame with GFP)
3 x G linker
Fc1

ACCAAAGGATGTCTTGACGATTACGCTTACCCCGAAAGTTACGTGTGTTGTTGTTGACATCAGTA   1040
TGGTTTCCTACAGAACTGCTAATGCGAATGGGGCTTTCAATGCACACAACAACAACTGTAGTCAT
P K D V L T I T L T P K V T C V V V D I S
(in frame with GFP)
Fc1

AAGATGACCCTGAAGTTCAATTTTCTTGGTTCGTTGACGATGTCGAAGTTCATACGGCTCAAACG   1105
TTCTACTGGGACTTCAAGTTAAAAGAACCAAGCAACTGCTACAGCTTCAAGTATGCCGAGTTTGC
K D D P E V Q F S W F V D D V E V H T A Q T
(in frame with GFP)
Fc1

CAACCTAGAGAGGAGCAATTTAATTCCACCTTTCGATCAGTCAGTGAACTCCCCATCATGCACCA   1170
GTTGGATCTCTCCTCGTTAAATTAAGGTGGAAAGCTAGTCAGTCACTTGAGGGGTAGTACGTGGT
Q P R E E Q F N S T F R S V S E L P I M H Q
(in frame with GFP)
Fc1

AGACTGGCTTAACGGGAAAGAGTTCAAATGCCGCGTAAACTCAGCAGCTTTCCCCGCCCCTATCG   1235
TCTGACCGAATTGCCCTTTCTCAAGTTTACGGCGCATTTGAGTCGTCGAAAGGGGCGGGGATAGC
D W L N G K E F K C R V N S A A F P A P I
(in frame with GFP)
Fc1
```

```
AAAAGACCATCTCTAAAACGAAGGGTAGGCCCAAAGCTCCACAAGTTTATACGATCCCCCCTCCC    1300
TTTTCTGGTAGAGATTTTGCTTCCCATCCGGGTTTCGAGGTGTTCAAATATGCTAGGGGGGAGGG
 E  K  T  I  S  K  T  K  G  R  P  K  A  P  Q  V  Y  T  I  P  P  P
(in frame with GFP)
Fc1

AAAGAGCAAATGGCTAAAGATAAAGTCTCGTTAACGTGCATGATCACAGATTTTTTTCCCGAGGA    1365
TTTCTCGTTTACCGATTTCTATTTCAGAGCAATTGCACGTACTAGTGTCTAAAAAAAGGGCTCCT
 K  E  Q  M  A  K  D  K  V  S  L  T  C  M  I  T  D  F  F  P  E  D
(in frame with GFP)
Fc1

CATCACGGTTGAGTGGCAATGGAATGGTCAACCTGCTGAAAACTATAAAAACACGCAACCTATTA    1430
GTAGTGCCAACTCACCGTTACCTTACCAGTTGGACGACTTTTGATATTTTTGTGCGTTGGATAAT
 I  T  V  E  W  Q  W  N  G  Q  P  A  E  N  Y  K  N  T  Q  P  I
(in frame with GFP)
Fc1

TGGATACGGACGGGTCGTATTTTGTGTACTCTAAGCTAAATGTaCAAAAGTCCAACTGGGAAGCG    1495
ACCTATGCCTGCCCAGCATAAAACACATGAGATTCGATTTACAtGTTTTCAGGTTGACCCTTCGC
 M  D  T  D  G  S  Y  F  V  Y  S  K  L  N  V  Q  K  S  N  W  E  A
(in frame with GFP)
Fc1

GGTAACACGTTTACATGTTCTGTACTGCACGAGGGTTTGCATAACCATCATACAGAGAAATCACT    1560
CCATTGTGCAAATGTACAAGACATGACGTGCTCCCAAACGTATTGGTAGTATGTCTCTTTAGTGA
 G  N  T  F  T  C  S  V  L  H  E  G  L  H  N  H  H  T  E  K  S  L
(in frame with GFP)
Fc1

TAGCCATAGTCCAGGGAAATAAatatagACGCGTTCAATTTAAACACCACCATCATCACCATCAT    1625
ATCGGTATCAGGTCCCTTTATTtatatcTGCGCAAGTTAAATTTGTGGTGGTAGTAGTGGTAGTA
 S  H  S  P  G  K  *
(in frame with GFP)                  This/thr
Fc1                                  his att

CCTGACTAGaCTTTCAGGCGATGTGTGCTGGAAGACATTCgGATCTTCCAGTGGTGCATGAACGC    1690
GGACTGATCtGAAAGTCCGCTACACACGACCTTCTGTAAGcCTAGAAGGTCACCACGTACTTGCG
This/thr
his att

ATGAGAAAGCCCCCGGAAGATCATCTTCCGGGGGCTTTTTTTTTGGCGCGTGACGCGTACAGGAA    1755
TACTCTTTCGGGGGCCTTCTAGTAGAAGGCCCCCGAAAAAAAAACCGCGCACTGCGCATGTCCTT
This/thr
his att                                          thr att

ACACAGAAAAAAGCCCGCACCTGACAGTGCGGGCTTTTTTTTTCGACCAAAGGTAACGAGGTAAC    1820
TGTGTCTTTTTTCGGGCGTGGACTGTCACGCCCGAAAAAAAAAGCTGGTTTCCATTGCTCCATTG
This/thr
thr att
```

HindIII ***End*** (1835)

A A C C A T G C G a a g c t t 3′

1835

T T G G T A C G C t t c g a a 5′

This/thr

thr att

Sequence: TVV3 ins annot proIGF1-FC1NC .dna (Linear / 1427 bp)
Enzymes: Unique 6+ Cutters (46 of 678 total)
Features: 8 total

Supplementary Figure 3C

Unique Cutters **Bold**

Start (0)

SacI

```
5' GagctcGCTCCCCCGCCGTCGTTCAATGAGAATGGATAAGAGGCTCGTGGGATTGACGTGAGGGG
3' CtcgagCGAGGGGGCGGCAGCAAGTTACTCTTACCTATTCTCCGAGCACCCTAACTGCACTCCCC
```

Prrn — 65

```
GCAGGGATGGCTATATTTCTGGGAGTTACTTCTACCCGATAGAGCTTAGAAGTTGGAAGTAATAA
CGTCCCTACCGATATAAAGACCCTCAATGAAGATGGGCTATCTCGAATCTTCAACCTTCATTATT
```

Prrn | Zm atpH 5' UTR — 130

Prrn TSS

```
TTTCTTGGTTGATTGTAtcCTTAACCATTTCTTTTTTTTTGACACGAGGAACTCATCATGACCCT
AAAGAACCAACTAACATagGAATTGGTAAAGAAAAAAAAACTGTGCTCCTTGAGTAGTACTGGGA
```

1: M T L

Zm atpH 5' UTR | proIGF1 — 195

```
TTGTGGTGCTGAACTGGTTGACGCTTTACAATTTGTCTGCGGTCCCAGGGGTTTTTATTTCAACA
AACACCACGACTTGACCAACTGCGAAATGTTAAACAGACGCCAGGGTCCCCAAAAATAAAGTTGT
```

5, 10, 15, 20: C G A E L V D A L Q F V C G P R G F Y F N

proIGF1 — 260

```
AGCCCACGGGTTATGGTAGTTCTATTCGAAGGGCACCACAAACGGGGATCGTGGATGAGTGTTGT
TCGGGTGCCCAATACCATCAAGATAAGCTTCCCGTGGTGTTTGCCCCTAGCACCTACTCACAACA
```

25, 30, 35, 40, 45: K P T G Y G S S I R R A P Q T G I V D E C C

proIGF1 — 325

```
TTTCGCTCGTGCGATCTTCGACGCCTAGAGATGTATTGTGCTCCCTTGAAACCGACGGGGGCCGC
AAAGCGAGCACGCTAGAAGCTGCGGATCTCTACATAACACGAGGGAACTTTGGCTGCCCCCGGCG
```

50, 55, 60, 65: F R S C D L R R L E M Y C A P L K P T G A A

proIGF1 — 390

```
GCGCTCAATCGCTGCTCAAGCTCACACAGACATGCCTAAAACGCAAAAAGAGGTTCATCTCAAAA
CGCGAGTTAGCGACGAGTTCGAGTGTGTCTGTACGGATTTTGCGTTTTTCTCCAAGTAGAGTTTT
```

70, 75, 80, 85: R S I A A Q A H T D M P K T Q K E V H L K

proIGF1 — 455

```
ATACGTCTCGCGGTAGCGCTGGTAACAAGACGTACCGAATGgggggggtGGTTGCAAGCCCTGT
TATGCAGAGCGCCATCGCGACCATTGTTCTGCATGGCTTACcccccccaCCAACGTTCGGGACA
```

90, 95, 100: N T S R G S A G N K T Y R M — G G G G C K P C (in frame with proIGF1)

proIGF1

3xG linker NC | Fc1 — 520

```
ATTTGTACGGTaCCGGAAGTATCTTCTGTGTTCATTTTTCCCCCTAAACCAAAGGATGTCTTGAC
TAAACATGCCAtGGCCTTCATAGAAGACACAAGTAAAAAGGGGGATTTGGTTTCCTACAGAACTG
```

I C T V P E V S S V F I F P P K P K D V L T (in frame with proIGF1)

Fc1 — 585

```
GATTACGCTTACCCCGAAAGTTACGTGTGTTGTTGTTGACATCAGTAAAGATGACCCTGAAGTTC     650
CTAATGCGAATGGGGCTTTCAATGCACACAACAACAACTGTAGTCATTTCTACTGGGACTTCAAG
 I  T  L  T  P  K  V  T  C  V  V  V  D  I  S  K  D  D  P  E  V
(in frame with proIGF1)
Fc1

AATTTTCTTGGTTCGTTGACGATGTCGAAGTTCATACGGCTCAAACGCAACCTAGAGAGGAGCAA     715
TTAAAAGAACCAAGCAACTGCTACAGCTTCAAGTATGCCGAGTTTGCGTTGGATCTCTCCTCGTT
Q  F  S  W  F  V  D  D  V  E  V  H  T  A  Q  T  Q  P  R  E  E  Q
(in frame with proIGF1)
Fc1

TTTAATTCCACCTTTCGATCAGTCAGTGAACTCCCCATCATGCACCAAGACTGGCTTAACGGGAA     780
AAATTAAGGTGGAAAGCTAGTCAGTCACTTGAGGGGTAGTACGTGGTTCTGACCGAATTGCCCTT
 F  N  S  T  F  R  S  V  S  E  L  P  I  M  H  Q  D  W  L  N  G  K
(in frame with proIGF1)
Fc1

AGAGTTCAAATGCCGCGTAAACTCAGCAGCTTTCCCCGCCCCTATCGAAAAGACCATCTCTAAAA     845
TCTCAAGTTTACGGCGCATTTGAGTCGTCGAAAGGGGCGGGGATAGCTTTTCTGGTAGAGATTTT
 E  F  K  C  R  V  N  S  A  A  F  P  A  P  I  E  K  T  I  S  K
(in frame with proIGF1)
Fc1

CGAAGGGTAGGCCCAAAGCTCCACAAGTTTATACGATCCCCCCTCCCAAAGAGCAAATGGCTAAA     910
GCTTCCCATCCGGGTTTCGAGGTGTTCAAATATGCTAGGGGGGAGGGTTTCTCGTTTACCGATTT
T  K  G  R  P  K  A  P  Q  V  Y  T  I  P  P  P  K  E  Q  M  A  K
(in frame with proIGF1)
Fc1

GATAAAGTCTCGTTAACGTGCATGATCACAGATTTTTTTCCCGAGGACATCACGGTTGAGTGGCA     975
CTATTTCAGAGCAATTGCACGTACTAGTGTCTAAAAAAAGGGCTCCTGTAGTGCCAACTCACCGT
 D  K  V  S  L  T  C  M  I  T  D  F  F  P  E  D  I  T  V  E  W  Q
(in frame with proIGF1)
Fc1

ATGGAATGGTCAACCTGCTGAAAACTATAAAAACACGCAACCTATTATGGATACGGACGGGTCGT     1040
TACCTTACCAGTTGGACGACTTTTGATATTTTTGTGCGTTGGATAATACCTATGCCTGCCCAGCA
 W  N  G  Q  P  A  E  N  Y  K  N  T  Q  P  I  M  D  T  D  G  S
(in frame with proIGF1)
Fc1

ATTTTGTGTACTCTAAGCTAAATGTaCAAAAGTCCAACTGGGAAGCGGGTAACACGTTTACATGT     1105
TAAAACACATGAGATTCGATTTACAtGTTTTCAGGTTGACCCTTCGCCCATTGTGCAAATGTACA
Y  F  V  Y  S  K  L  N  V  Q  K  S  N  W  E  A  G  N  T  F  T  C
(in frame with proIGF1)
Fc1
```

```
T C T G T A C T G C A C G A G G G T T T G C A T A A C C A T C A T A C A G A G A A A T C A C T T A G C C A T A G T C C A G G G A A   1170
A G A C A T G A C G T G C T C C C A A A C G T A T T G G T A G T A T G T C T C T T T A G T G A A T C G G T A T C A G G T C C C T T
 S   V   L   H   E   G   L   H   N   H   H   T   E   K   S   L   S   H   S   P   G   K
(in frame with proIGF1)
Fc1

A T A A a t a t a g A C G C G T T C A A T T T A A A C A C C A C C A T C A T C A C C A T C A T C C T G A C T A G a C T T T C A G G   1235
T A T T t a t a t c T G C G C A A G T T A A A T T T G T G G T G G T A G T A G T G G T A G T A G G A C T G A T C t G A A A G T C C
*
This att
Fc1

C G A T G T G T G C T G G A A G A C A T T C g G A T C T T C C A G T G G T G C A T G A A C G C A T G A G A A A G C C C C C G G A A   1300
G C T A C A C A C G A C C T T C T G T A A G c C T A G A A G G T C A C C A C G T A C T T G C G T A C T C T T T C G G G G G C C T T
This att

G A T C A T C T T C C G G G G G C T T T T T T T T G G C G C G T G A C G C G T A C A G G A A A C A C A G A A A A A A G C C C G C   1365
C T A G T A G A A G G C C C C C G A A A A A A A A C C G C G C A C T G C G C A T G T C C T T T G T G T C T T T T T T C G G G C G
This att                                                    thr att

                                                                            HindIII      End (1427)
A C C T G A C A G T G C G G G C T T T T T T T T T C G A C C A A A G G T A A C G A G G T A A C A A C C A T G C G a a g c t t   3′
                                                                                                            1427
T G G A C T G T C A C G C C C G A A A A A A A A A G C T G G T T T C C A T T G C T C C A T T G T T G G T A C G C t t c g a a   5′
thr att
```

	Feature	Location	Size			Type
✓	**Prrn**	7 .. 90	84 bp		⟼	misc_feature
	/note = rrn operon promoter					
✓	**Prrn TSS**	90 .. 90	1 bp		⟼	misc_feature
	/note = rrn promoter transcription start site					
✓	**Zm atpH 5' UTR**	91 .. 187	97 bp		⟼	misc_feature
✓	**proIGF1**	188 .. 496	309 bp		→	CDS
	/translation = MTLCGAELVDALQFVCGPRGFYFNKPTGYGSSIRRAPQTGIVDECCFRSCDLRRLEMYCAPLKPTGAARSIAAQAHTDMPKTQKEVHLKNTSRGSAGNKTYRM<br>103 amino acids = 11,4 kDa					
✓	**3xG linker NC**	497 .. 505	9 bp		⟼	misc_feature
✓	**Fc1**	506 .. 1171	666 bp		⟼	misc_feature
✓	**This att**	1181 .. 1338	158 bp		⟼	misc_feature
	/note = histidine attenuator					
✓	**thr att**	1339 .. 1421	83 bp		⟼	misc_feature
	/note = threonine attenuator					

Sequence: TVV4 ins C annot.dna (Linear / 1457 bp)
Enzymes: Unique 6+ Cutters (45 of 678 total)
Features: 8 total

Supplementary Figure 3D

Unique Cutters **Bold**

Start (0)

SacI

```
5′ GagctcGCTCCCCCGCCGTCGTTCAATGAGAATGGATAAGAGGCTCGTGGGATTGACGTGAGGGG   65
3′ CtcgagCGAGGGGGCGGCAGCAAGTTACTCTTACCTATTCTCCGAGCACCCTAACTGCACTCCCC
```

Prrn

```
GCAGGGATGGCTATATTTCTGGGAGTTACTTCTACCCGATAGAGCTTAGAAGTTGGAAGTAATAA   130
CGTCCCTACCGATATAAAGACCCTCAATGAAGATGGGCTATCTCGAATCTTCAACCTTCATTATT
```

Prrn | Zm atpH 5'-UTR

Prrn TSS

```
TTTCTTGGTTGATTGTAtcCTTAACCATTTCTTTTTTTTTTGACACGAGGAACTCATCATGGGGCC   195
AAAGAACCAACTAACATagGAATTGGTAAAGAAAAAAAAAACTGTGCTCCTTGAGTAGTACCCCGG
```

1: M G P

Zm atpH 5'-UTR | proIGF1

```
TGAAACCCTTTGTGGTGCTGAACTGGTTGACGCTTTACAATTTGTCTGCGGTCCCAGGGGTTTTT   260
ACTTTGGGAAACACCACGACTTGACCAACTGCGAAATGTTAAACAGACGCCAGGGTCCCCAAAAA
```

5, 10, 15, 20: E T L C G A E L V D A L Q F V C G P R G F

proIGF1

```
ATTTCAACAAGCCCACGGGTTATGGTAGTTCTATTCGAAGGGCACCACAAACGGGGATCGTGGAT   325
TAAAGTTGTTCGGGTGCCCAATACCATCAAGATAAGCTTCCCGTGGTGTTTGCCCCTAGCACCTA
```

25, 30, 35, 40, 45: Y F N K P T G Y G S S I R R A P Q T G I V D

proIGF1

```
GAGTGTTGTTTTCGCTCGTGCGATCTTCGACGCCTAGAGATGTATTGTGCTCCCTTGAAACCGAC   390
CTCACAACAAAAGCGAGCACGCTAGAAGCTGCGGATCTCTACATAACACGAGGGAACTTTGGCTG
```

50, 55, 60, 65: E C C F R S C D L R R L E M Y C A P L K P T

proIGF1

```
GGGGGCCGCGCGCTCAATCGCTGCTCAAGCTCACACAGACATGCCTAAAACGCAAAAAGAGGTTC   455
CCCCCGGCGCGCGAGTTAGCGACGAGTTCGAGTGTGTCTGTACGGATTTTGCGTTTTTCTCCAAG
```

70, 75, 80, 85: G A A R S I A A Q A H T D M P K T Q K E V

proIGF1

```
ATCTCAAAAATACGTCTCGCGGTAGCGCTGGTAACAAGACGTACCGAATGGGGCGATCCAGACGA   520
TAGAGTTTTTATGCAGAGCGCCATCGCGACCATTGTTCTGCATGGCTTACCCCGCTAGGTCTGCT
```

90, 95, 100, 105: H L K N T S R G S A G N K T Y R M | G R S R R (in frame with proIGF1)

proIGF1

2x furin cleavage site

```
GGTCGATCTCGAAGAGGTTGCAAGCCCTGTATTTGTACGGTaCCGGAAGTATCTTCTGTGTTCAT   585
CCAGCTAGAGCTTCTCCAACGTTCGGGACATAAACATGCCAtGGCCTTCATAGAAGACACAAGTA
```

G R S R R G C K P C I C T V P E V S S V F I (in frame with proIGF1)

2x furin cleavage site | Fc1

T T T T C C C C C T A A A C C A A A G G A T G T C T T G A C G A T T A C G C T T A C C C C G A A A G T T A C G T G T G T T G T T G
650
A A A A G G G G G A T T T G G T T T C C T A C A G A A C T G C T A A T G C G A A T G G G G C T T T C A A T G C A C A C A A C A A C
F P P K P K D V L T I T L T P K V T C V V
(in frame with proIGF1)
Fc1

T T G A C A T C A G T A A A G A T G A C C C T G A A G T T C A A T T T T C T T G G T T C G T T G A C G A T G T C G A A G T T C A T
715
A A C T G T A G T C A T T T C T A C T G G G A C T T C A A G T T A A A A G A A C C A A G C A A C T G C T A C A G C T T C A A G T A
V D I S K D D P E V Q F S W F V D D V E V H
(in frame with proIGF1)
Fc1

A C G G C T C A A A C G C A A C C T A G A G A G G A G C A A T T T A A T T C C A C C T T T C G A T C A G T C A G T G A A C T C C C
780
T G C C G A G T T T G C G T T G G A T C T C T C C T C G T T A A A T T A A G G T G G A A A G C T A G T C A G T C A C T T G A G G G
T A Q T Q P R E E Q F N S T F R S V S E L P
(in frame with proIGF1)
Fc1

C A T C A T G C A C C A A G A C T G G C T T A A C G G G A A A G A G T T C A A A T G C C G C G T A A A C T C A G C A G C T T T C C
845
G T A G T A C G T G G T T C T G A C C G A A T T G C C C T T T C T C A A G T T T A C G G C G C A T T T G A G T C G T C G A A A G G
I M H Q D W L N G K E F K C R V N S A A F
(in frame with proIGF1)
Fc1

C C G C C C C T A T C G A A A A G A C C A T C T C T A A A A C G A A G G G T A G G C C C A A A G C T C C A C A A G T T T A T A C G
910
G G C G G G G A T A G C T T T T C T G G T A G A G A T T T T G C T T C C C A T C C G G G T T T C G A G G T G T T C A A A T A T G C
P A P I E K T I S K T K G R P K A P Q V Y T
(in frame with proIGF1)
Fc1

A T C C C C C C T C C C A A A G A G C A A A T G G C T A A A G A T A A A G T C T C G T T A A C G T G C A T G A T C A C A G A T T T
975
T A G G G G G G A G G G T T T C T C G T T T A C C G A T T T C T A T T T C A G A G C A A T T G C A C G T A C T A G T G T C T A A A
I P P P K E Q M A K D K V S L T C M I T D F
(in frame with proIGF1)
Fc1

T T T T C C C G A G G A C A T C A C G G T T G A G T G G C A A T G G A A T G G T C A A C C T G C T G A A A A C T A T A A A A A C A
1040
A A A A G G G C T C C T G T A G T G C C A A C T C A C C G T T A C C T T A C C A G T T G G A C G A C T T T T G A T A T T T T T G T
F P E D I T V E W Q W N G Q P A E N Y K N
(in frame with proIGF1)
Fc1

C G C A A C C T A T T A T G G A T A C G G A C G G G T C G T A T T T T G T G T A C T C T A A G C T A A A T G T a C A A A A G T C C
1105
G C G T T G G A T A A T A C C T A T G C C T G C C C A G C A T A A A A C A C A T G A G A T T C G A T T T A C A t G T T T T C A G G
T Q P I M D T D G S Y F V Y S K L N V Q K S
(in frame with proIGF1)
Fc1

```
A A C T G G G A A G C G G G T A A C A C G T T T A C A T G T T C T G T A C T G C A C G A G G G T T T G C A T A A C C A T C A T A C   1170
T T G A C C C T T C G C C C A T T G T G C A A A T G T A C A A G A C A T G A C G T G C T C C C A A A C G T A T T G G T A G T A T G
  N     W     E     A     G     N     T     F     T     C     S     V     L     H     E     G     L     H     N     H     H     T
                                   (in frame with proIGF1)
Fc1

A G A G A A A T C A C T T A G C C A T A G T C C A G G G A A A T A A a t a t a g A C G C G T T C A A T T T A A A C A C C A C C A T   1235
T C T C T T T A G T G A A T C G G T A T C A G G T C C C T T T A T T t a t a t c T G C G C A A G T T A A A T T T G T G G T G G T A
  E     K     S     L     S     H     S     P     G     K     *
         (in frame with proIGF1)                                        This att
Fc1

C A T C A C C A T C A T C C T G A C T A G a C T T T C A G G C G A T G T G T G C T G G A A G A C A T T C g G A T C T T C C A G T G   1300
G T A G T G G T A G T A G G A C T G A T C t G A A A G T C C G C T A C A C A C G A C C T T C T G T A A G c C T A G A A G G T C A C
This att

G T G C A T G A A C G C A T G A G A A A G C C C C C G G A A G A T C A T C T T C C G G G G G C T T T T T T T T T G G C G C G T G A   1365
C A C G T A C T T G C G T A C T C T T T C G G G G G C C T T C T A G T A G A A G G C C C C C G A A A A A A A A A C C G C G C A C T
This att

C G C G T A C A G G A A A C A C A G A A A A A A G C C C G C A C C T G A C A G T G C G G G C T T T T T T T T T C G A C C A A A G G   1430
G C G C A T G T C C T T T G T G T C T T T T T T C G G G C G T G G A C T G T C A C G C C C G A A A A A A A A A G C T G G T T T C C
thr att
This att

                    HindIII     End (1457)
T A A C G A G G T A A C A A C C A T G C G a a g c t t   3'
                                                          1457
A T T G C T C C A T T G T T G G T A C G C t t c g a a   5'
thr att
```

	Feature		Location	Size			Type
✓	**Prrn**		7 .. 90	84 bp			misc_feature
	/note	= rrn operon promoter					
✓	**Prrn TSS**		90 .. 90	1 bp			misc_feature
	/note	= Prrn promoter trasncription start site					
✓	**Zm atpH 5'-UTR**		91 .. 187	97 bp			misc_feature
✓	**proIGF1**		188 .. 505	318 bp		→	CDS
	/translation	= MGPETLCGAELVDALQFVCGPRGFYFNKPTGYGSSIRRAPQTGIVDECCFRSCDLRRLEMYCAPLKPTGAARSIAAQAHTDMPKTQKEVHLKNTSRGSAGNKTYRM<br>106 amino acids = 11,6 kDa					
✓	**2x furin cleavage site**		506 .. 535	30 bp			misc_feature
✓	**Fc1**		536 .. 1201	666 bp		→	misc_feature
✓	**This att**		1211 .. 1368	158 bp			misc_feature
	/note	= histidine attenuator					
✓	**thr att**		1369 .. 1451	83 bp			misc_feature
	/note	= threonine attenuator					

Sequence: TVV5 insert annot.dna (Linear / 1841 bp)
Enzymes: Unique 6+ Cutters (37 of 678 total)
Features: 8 total

Supplementary Figure 3E

Unique Cutters **Bold**

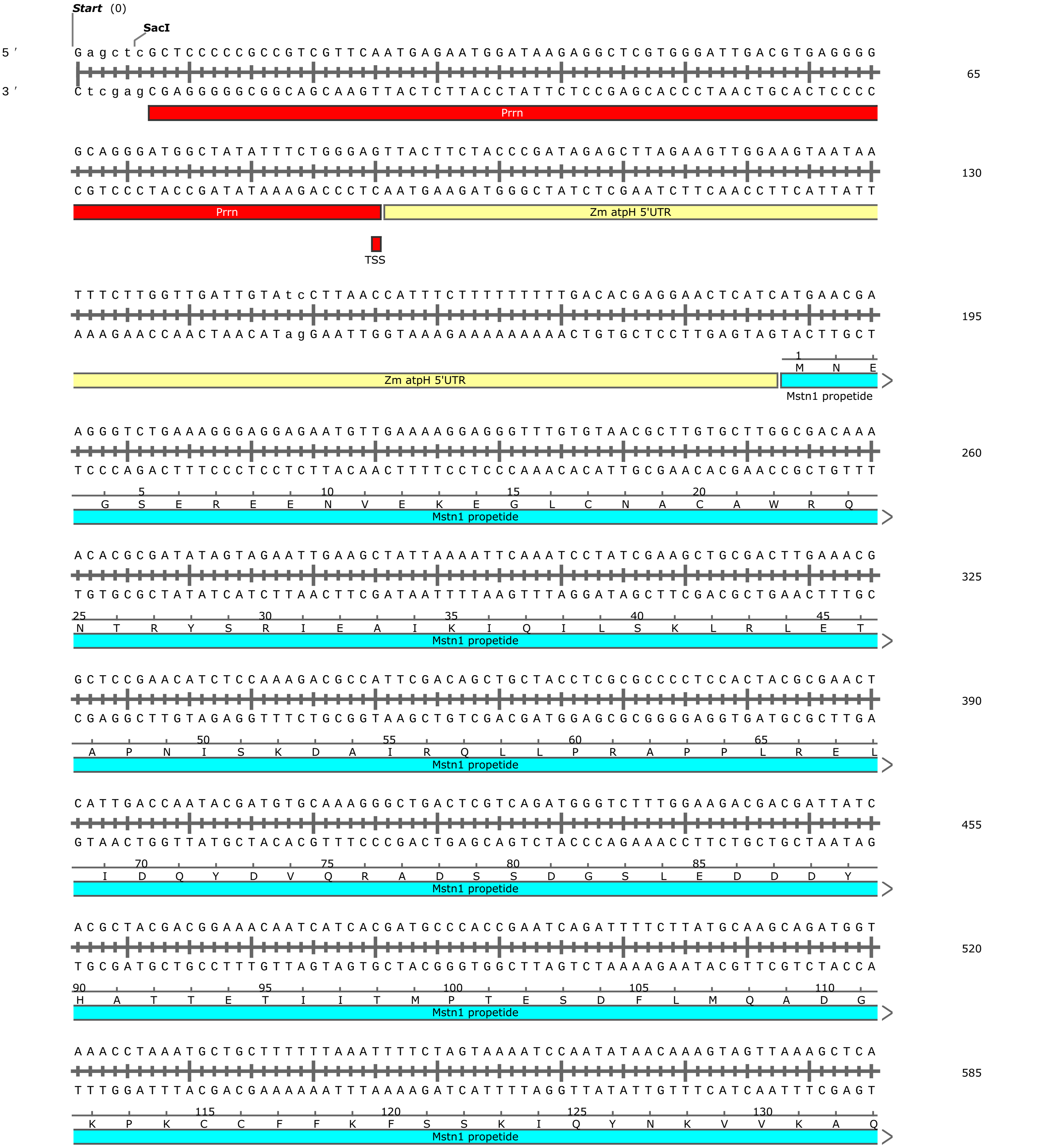

```
ACTTTGGATTTACTTACGCCCTGTCAAAACACCGACCACGGTATTCGTTCAAATCCTTAGACTAA   650
TGAAACCTAAATGAATGCGGGACAGTTTTGTGGCTGGTGCCATAAGCAAGTTTAGGAATCTGATT
135 140 145 150
L W I Y L R P V K T P T T V F V Q I L R L
Mstn1 propetide

TCAAACCCATGAAAGATGGGACGCGATATACCGGTATCCGCTCTTTGAAATTAGACATGAGCCCC   715
AGTTTGGGTACTTTCTACCCTGCGCTATATGGCCATAGGCGAGAAACTTTAATCTGTACTCGGGG
155 160 165 170 175
I K P M K D G T R Y T G I R S L K L D M S P
Mstn1 propetide

GGTACGGGGATCTGGCAATCCATTGATGTTAAAACGGTTCTCCAAAACTGGCTTAAGCAACCCGA   780
CCATGCCCCTAGACCGTTAGGTAACTACAATTTTGCCAAGAGGTTTTGACCGAATTCGTTGGGCT
180 185 190 195
G T G I W Q S I D V K T V L Q N W L K Q P E
Mstn1 propetide

ATCTAATCTTGGTATCGAGATTAAGGCTCTTGACGAAAACGGTCATGACCTGGCGGTGACATTCC   845
TAGATTAGAACCATAGCTCTAATTCCGAGAACTGCTTTTGCCAGTACTGGACCGCCACTGTAAGG
200 205 210 215
S N L G I E I K A L D E N G H D L A V T F
Mstn1 propetide

CTGGTCCCGGTGAAGACGGTTTGAATCCATTTctcGAGGTCAAGGTTACGGATACGCCGAAGGGG   910
GACCAGGGCCACTTCTGCCAAACTTAGGTAAAgagCTCCAGTTCCAATGCCTATGCGGCTTCCCC
220 225 230 235 240
P G P G E D G L N P F L E V K V T D T P K G
Mstn1 propetide
Linker

CTTGATGGTGGTTGCAAGCCCTGTATTTGTACGGTaCCGGAAGTATCTTCTGTGTTCATTTTTCC   975
GAACTACCACCAACGTTCGGGACATAAACATGCCAtGGCCTTCATAGAAGACACAAGTAAAAAGG
L D G G C K P C I C T V P E V S S V F I F P
(in frame with Mstn1 propetide)
Linker   Fc1

CCCTAAACCAAAGGATGTCTTGACGATTACGCTTACCCCGAAAGTTACGTGTGTTGTTGTTGACA   1040
GGGATTTGGTTTCCTACAGAACTGCTAATGCGAATGGGGCTTTCAATGCACACAACAACAACTGT
P K P K D V L T I T L T P K V T C V V V D
(in frame with Mstn1 propetide)
Fc1

TCAGTAAAGATGACCCTGAAGTTCAATTTTCTTGGTTCGTTGACGATGTCGAAGTTCATACGGCT   1105
AGTCATTTCTACTGGGACTTCAAGTTAAAAGAACCAAGCAACTGCTACAGCTTCAAGTATGCCGA
I S K D D P E V Q F S W F V D D V E V H T A
(in frame with Mstn1 propetide)
Fc1

CAAACGCAACCTAGAGAGGAGCAATTTAATTCCACCTTTCGATCAGTCAGTGAACTCCCCATCAT   1170
GTTTGCGTTGGATCTCTCCTCGTTAAATTAAGGTGGAAAGCTAGTCAGTCACTTGAGGGGTAGTA
Q T Q P R E E Q F N S T F R S V S E L P I M
(in frame with Mstn1 propetide)
Fc1
```

```
GCACCAAGACTGGCTTAACGGGAAAGAGTTCAAATGCCGCGTAAACTCAGCAGCTTTCCCCGCCC   1235
CGTGGTTCTGACCGAATTGCCCTTTCTCAAGTTTACGGCGCATTTGAGTCGTCGAAAGGGGCGGG
  H  Q  D  W  L  N  G  K  E  F  K  C  R  V  N  S  A  A  F  P  A
(in frame with Mstn1 propetide)
Fc1

CTATCGAAAAGACCATCTCTAAAACGAAGGGTAGGCCCAAAGCTCCACAAGTTTATACGATCCCC   1300
GATAGCTTTTCTGGTAGAGATTTTGCTTCCCATCCGGGTTTCGAGGTGTTCAAATATGCTAGGGG
P  I  E  K  T  I  S  K  T  K  G  R  P  K  A  P  Q  V  Y  T  I  P
(in frame with Mstn1 propetide)
Fc1

CCTCCCAAAGAGCAAATGGCTAAAGATAAAGTCTCGTTAACGTGCATGATCACAGATTTTTTTCC   1365
GGAGGGTTTCTCGTTTACCGATTTCTATTTCAGAGCAATTGCACGTACTAGTGTCTAAAAAAAGG
 P  P  K  E  Q  M  A  K  D  K  V  S  L  T  C  M  I  T  D  F  F  P
(in frame with Mstn1 propetide)
Fc1

CGAGGACATCACGGTTGAGTGGCAATGGAATGGTCAACCTGCTGAAAACTATAAAAACACGCAAC   1430
GCTCCTGTAGTGCCAACTCACCGTTACCTTACCAGTTGGACGACTTTTGATATTTTTGTGCGTTG
  E  D  I  T  V  E  W  Q  W  N  G  Q  P  A  E  N  Y  K  N  T  Q
(in frame with Mstn1 propetide)
Fc1

CTATTATGGATACGGACGGGTCGTATTTTGTGTACTCTAAGCTAAATGTaCAAAAGTCCAACTGG   1495
GATAATACCTATGCCTGCCCAGCATAAAACACATGAGATTCGATTTACAtGTTTTCAGGTTGACC
P  I  M  D  T  D  G  S  Y  F  V  Y  S  K  L  N  V  Q  K  S  N  W
(in frame with Mstn1 propetide)
Fc1

GAAGCGGGTAACACGTTTACATGTTCTGTACTGCACGAGGGTTTGCATAACCATCATACAGAGAA   1560
CTTCGCCCATTGTGCAAATGTACAAGACATGACGTGCTCCCAAACGTATTGGTAGTATGTCTCTT
 E  A  G  N  T  F  T  C  S  V  L  H  E  G  L  H  N  H  H  T  E  K
(in frame with Mstn1 propetide)
Fc1

ATCACTTAGCCATAGTCCAGGGAAATAAatatagACGCGTTCAATTTAAACACCACCATCATCAC   1625
TAGTGAATCGGTATCAGGTCCCTTTATTtatatcTGCGCAAGTTAAATTTGTGGTGGTAGTAGTG
  S  L  S  H  S  P  G  K  *
(in frame with Mstn1 propetide)                This att
Fc1

CATCATCCTGACTAGaCTTTCAGGCGATGTGTGCTGGAAGACATTCgGATCTTCCAGTGGTGCAT   1690
GTAGTAGGACTGATCtGAAAGTCCGCTACACACGACCTTCTGTAAGcCTAGAAGGTCACCACGTA
This att

GAACGCATGAGAAAGCCCCCGGAAGATCATCTTCCGGGGGCTTTTTTTTTGGCGCGTGACGCGTA   1755
CTTGCGTACTCTTTCGGGGGCCTTCTAGTAGAAGGCCCCCGAAAAAAAAACCGCGCACTGCGCAT
This att                                                       thr att
```

```
CAGGAAACACAGAAAAAAGCCCGCACCTGACAGTGCGGGCTTTTTTTTTCGACCAAAGGTAACGA          1820
GTCCTTTGTGTCTTTTTTCGGGCGTGGACTGTCACGCCCGAAAAAAAAAGCTGGTTTCCATTGCT
thr att

                       HindIII        End (1841)
GGTAACAACCATGCGaagctt        3′
                                   1841
CCATTGTTGGTACGCttcgaa        5′
thr att
```

	Feature			Location	Size			Type
✓	**Prrn**			7 .. 90	84 bp			misc_feature
	/note	=	tobacco rrn operon PEP Promoter					
✓	**TSS**			90 .. 90	1 bp			misc_feature
	/note	=	Prrn transcription start site					
✓	**Zm atpH 5'UTR**			91 .. 187	97 bp			misc_feature
✓	**Mstn1 propetide**			188 .. 907	720 bp		→	CDS
	/translation	=	MNEGSEREENVEKEGLCNACAWRQNTRYSRIEAIKIQILSKLRLETAPNISKDAIRQLLPRAPPLRELIDQYDVQRADSSDGSLEDDDYHATTETIITMPTESDFLMQADGKPKCCFFKFSSKIQYNKVVKAQLWIYLRPVKTPTTVFVQILRLIKPMKDGTRYTGIRSLKLDMSPGTGIWQSIDVKTVLQNWLKQPESNLGIEIKALDENGHDLAVTFPGPGEDGLNPFLEVKVTDTPK<br>240 amino acids = 27,2 kDa					
✓	**Linker**			908 .. 919	12 bp			misc_feature
	/note	=	RSRR to GLDG (D76A) mutation for BMP1 cleavage prevention					
✓	**Fc1**			920 .. 1585	666 bp			misc_feature
✓	**This att**			1595 .. 1752	158 bp			misc_feature
	/note	=	histidine attenuator					
✓	**thr att**			1753 .. 1835	83 bp			misc_feature
	/note	=	threonine attenuator					

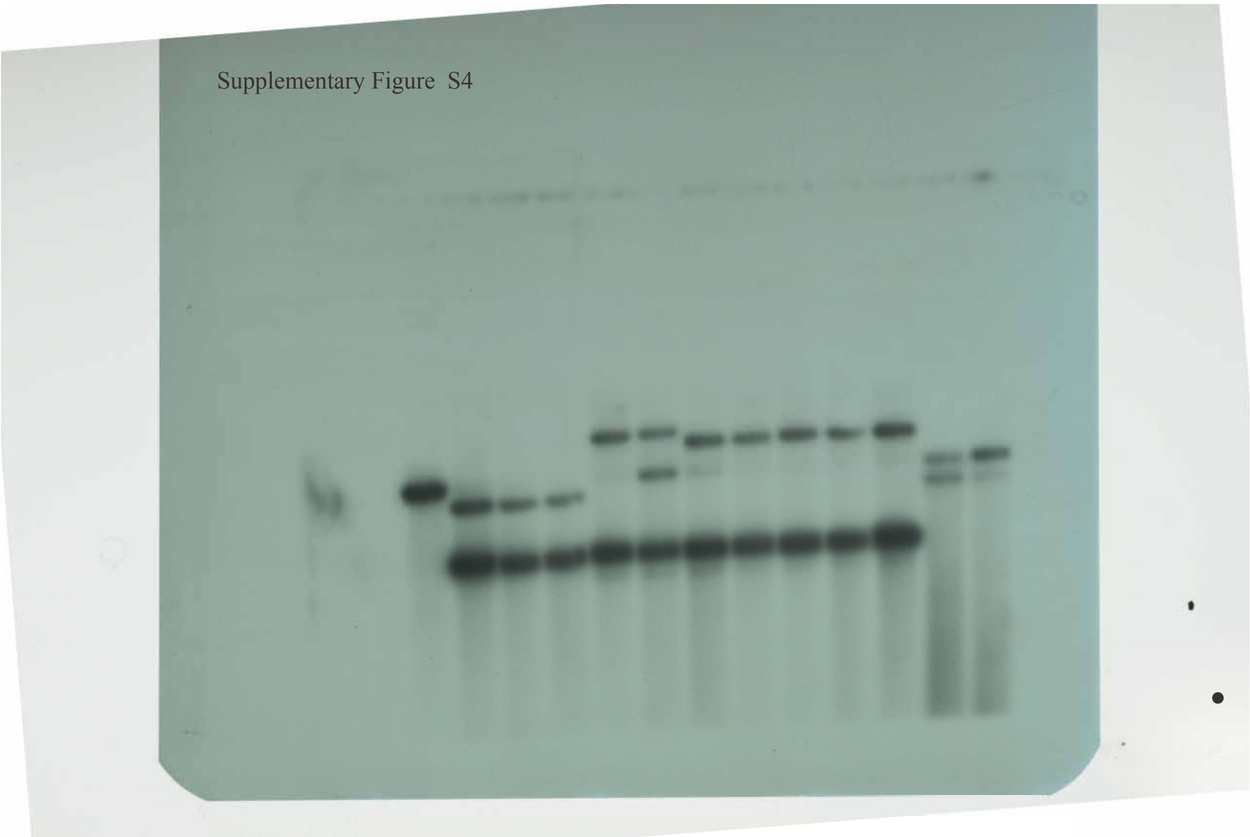

Supplementary Figure S4. Original Southern blot corresponding to Fig 1b, upper. The two lanes farthest to the right represent transplastomic lines derived from a construct not included in this journal article.

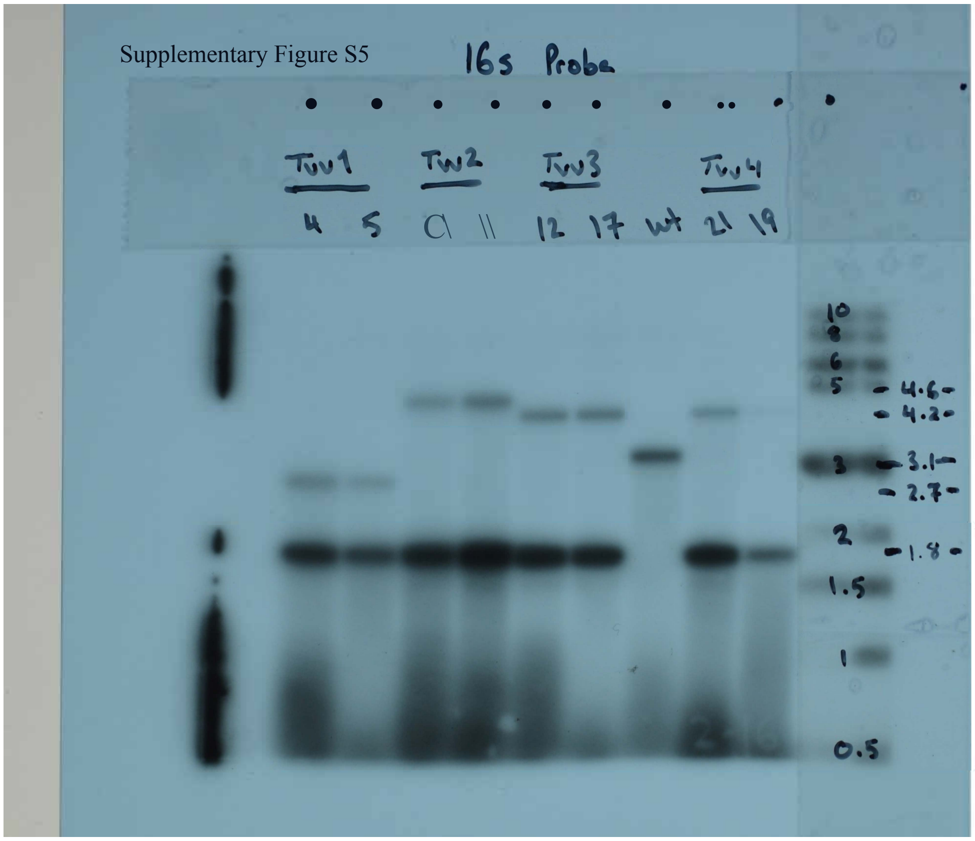

Supplementary Figure S5. Original Southern blot corresponding to Fig 1b, lower.

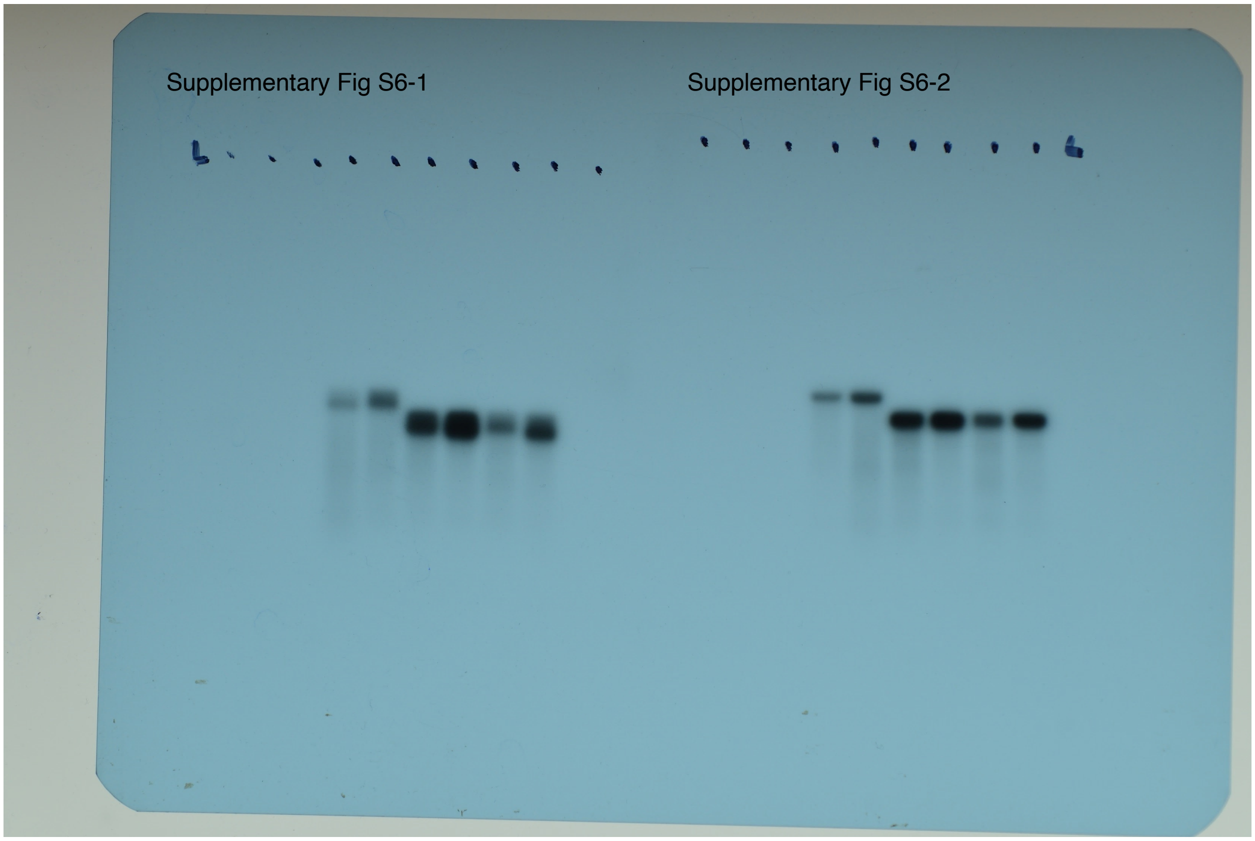

Supplementary Figure S6. Two replicates of the original Northern blot used to detect transcription of the transgenes in the transplastomic lines. Fig S6-2 is the original Northern blot corresponding to Fig 1c, upper.

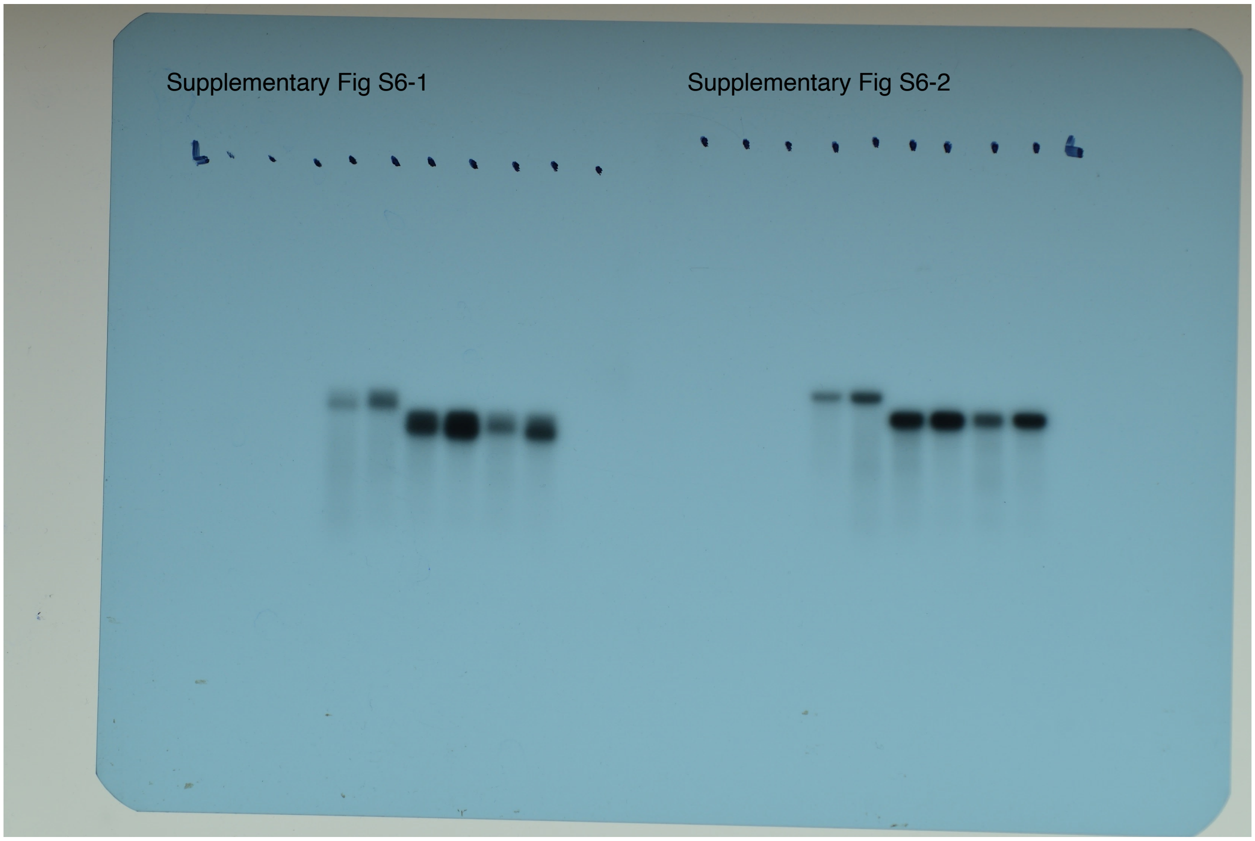

Supplementary Figure S6. Two replicates of the original Northern blot used to detect transcription of the transgenes in the transplastomic lines. Fig S6-2 is the original Northern blot corresponding to Fig 1c, upper.

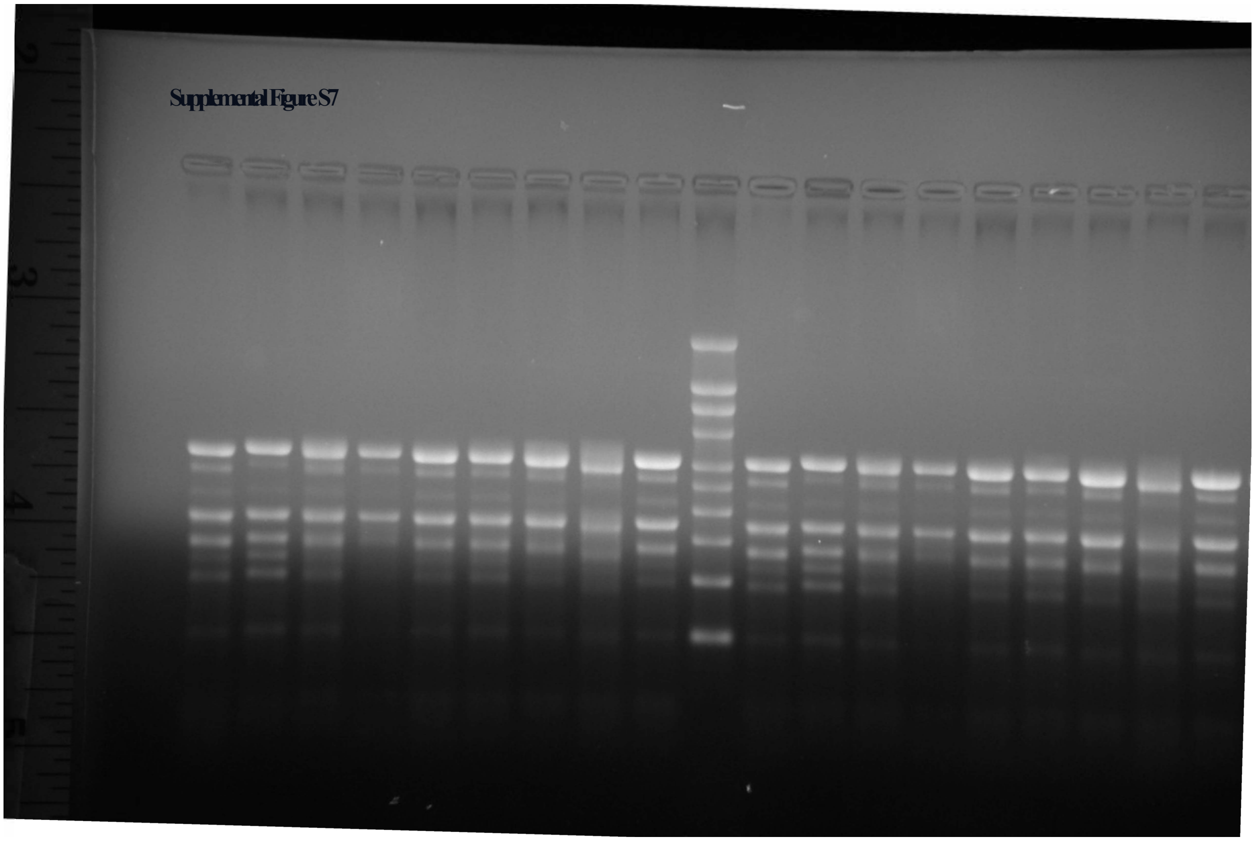

Supplementary Figure S7. Original agarose gel electrophoresis of RNA extracts used to generate the Northern blots in Fig 1c. Lanes 1 through 9 provide the image of the 28S in Fig 1c, upper. Lanes 11 through 19 provide the image of the 28S in Fig 1c, lower.

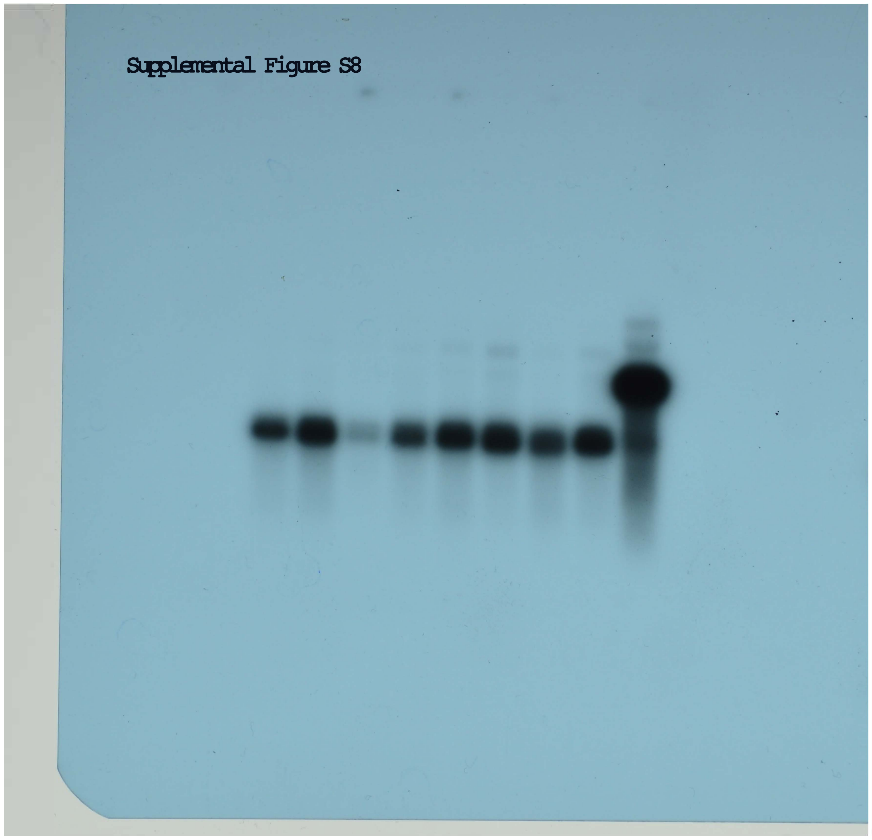

Supplementary Figure S8. Original Northern blot corresponding to Fig 1c, lower. The lane farthest to the right represents RNA extracts derived from the MRR13 transplastomic line.

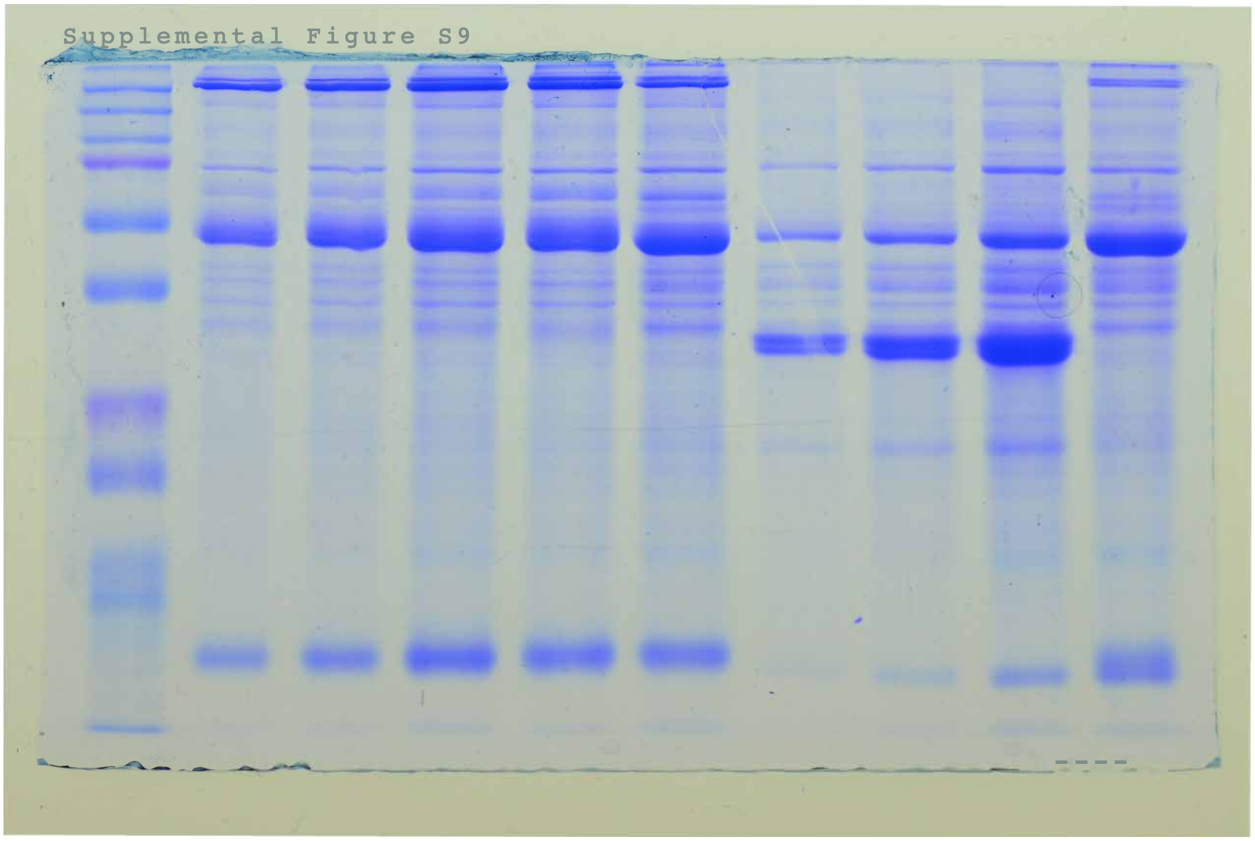

Supplementary Figure S9. Original Coomassie brilliant blue gel corresponding to Fig 2a.

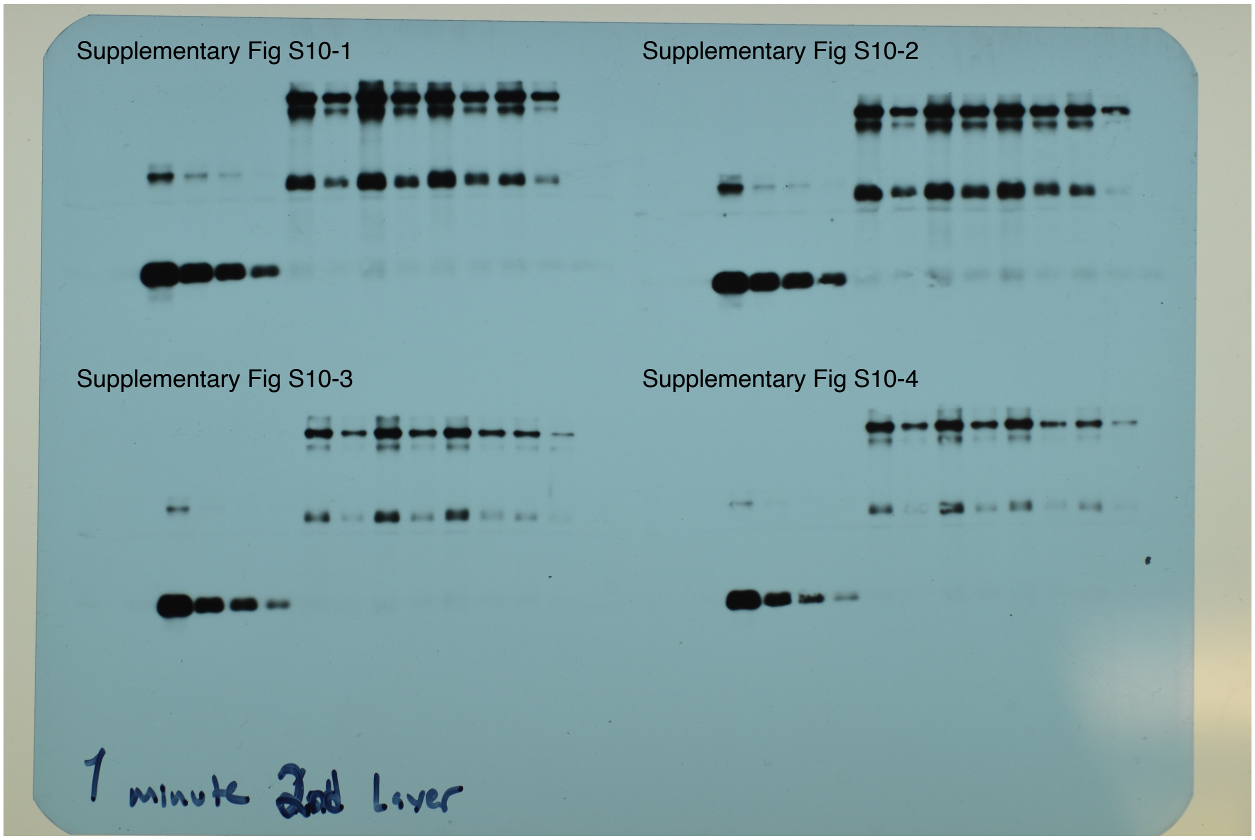

Supplementary Figure S10. Four replicates of the original immunoblots used to calculate the accumulation of GFP-Fc1 in TVV2 lines. The gels of the blots used to generate Fig S10-1 and S10-2 were loaded with double the protein in each lane compared to Fig S10-3 and S10-4. In each blot, a fourth TVV2 line was used to generate the bands in the last two lanes (14th and 15th lanes), however this line was not used in the calculations. Fig S10-3 is the original immunoblot corresponding to Fig 2b.

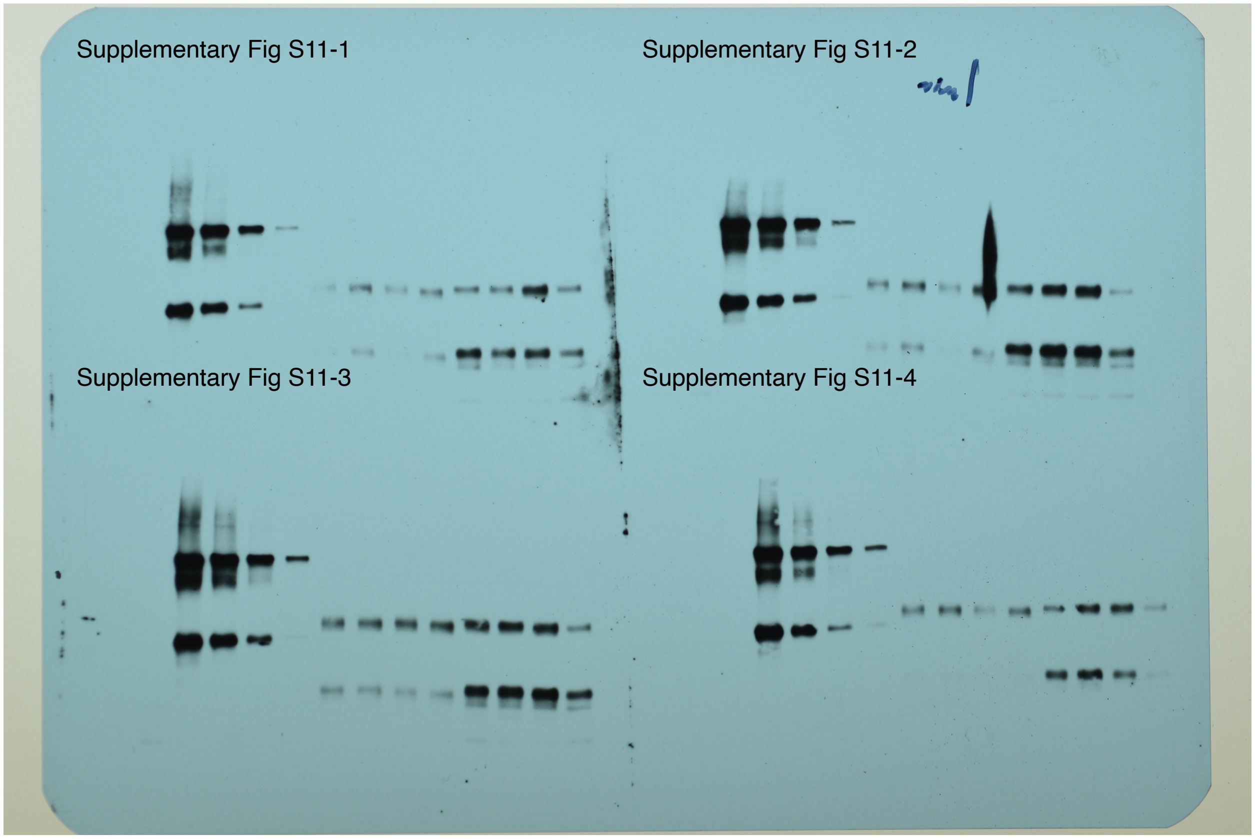

Supplementary Figure S11. Four replicates of the original immunoblots used to calculate the accumulation of proIGF1-Fc1NC and proIGF1-Fc1C proteins in TVV3 and TVV4 lines. Fig S11-3 is the original immunoblot corresponding to Fig 2c.

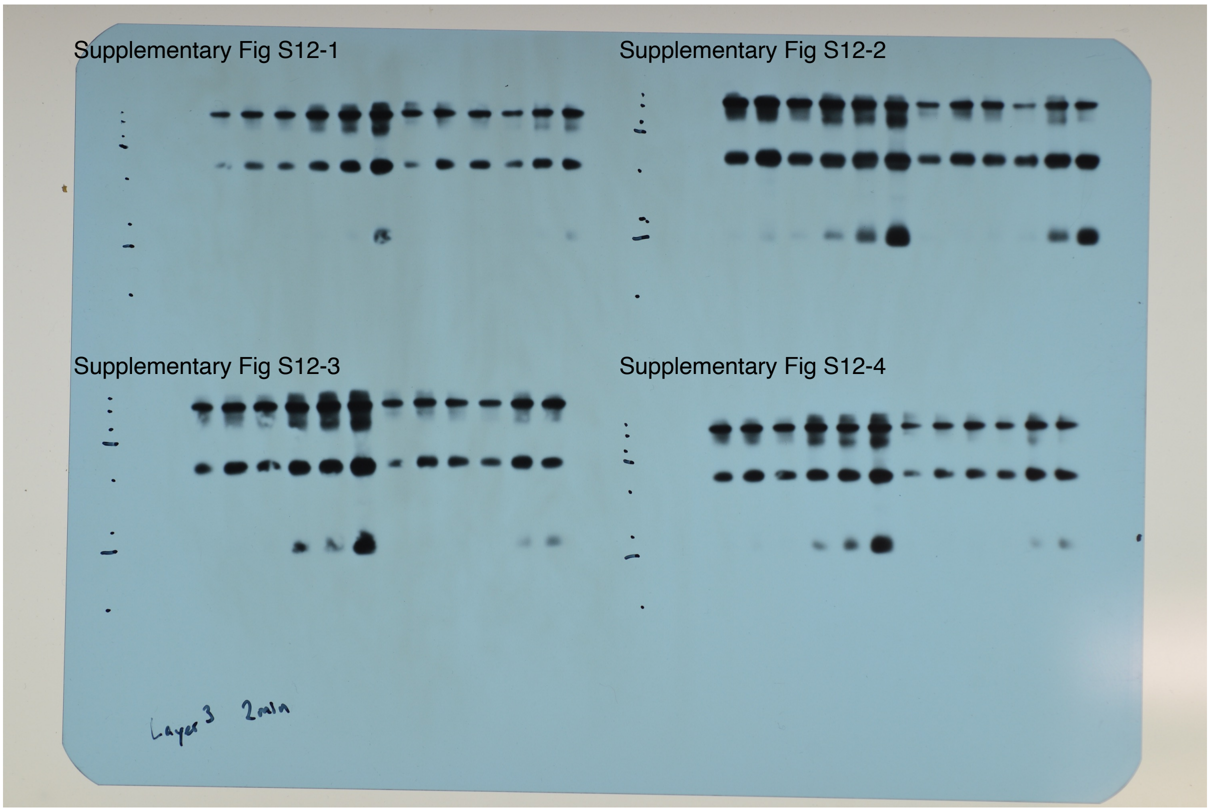

Supplementary Figure S12. Four replicates of the original immunoblots used to detect protein accumulation in the leaves of two TVV2 lines. The gel of the blot used to generate Fig S12-1 was loaded with 150 ng of protein from each leaf. The gel of the blot used to generate Fig S12-2 was loaded with 600 ng of protein from each leaf. The gels of the blots used to generate Fig S12-3 and S12-4 were loaded with 300 ng of protein from each leaf. Fig S12-1 is the original blot corresponding to Fig 3b.

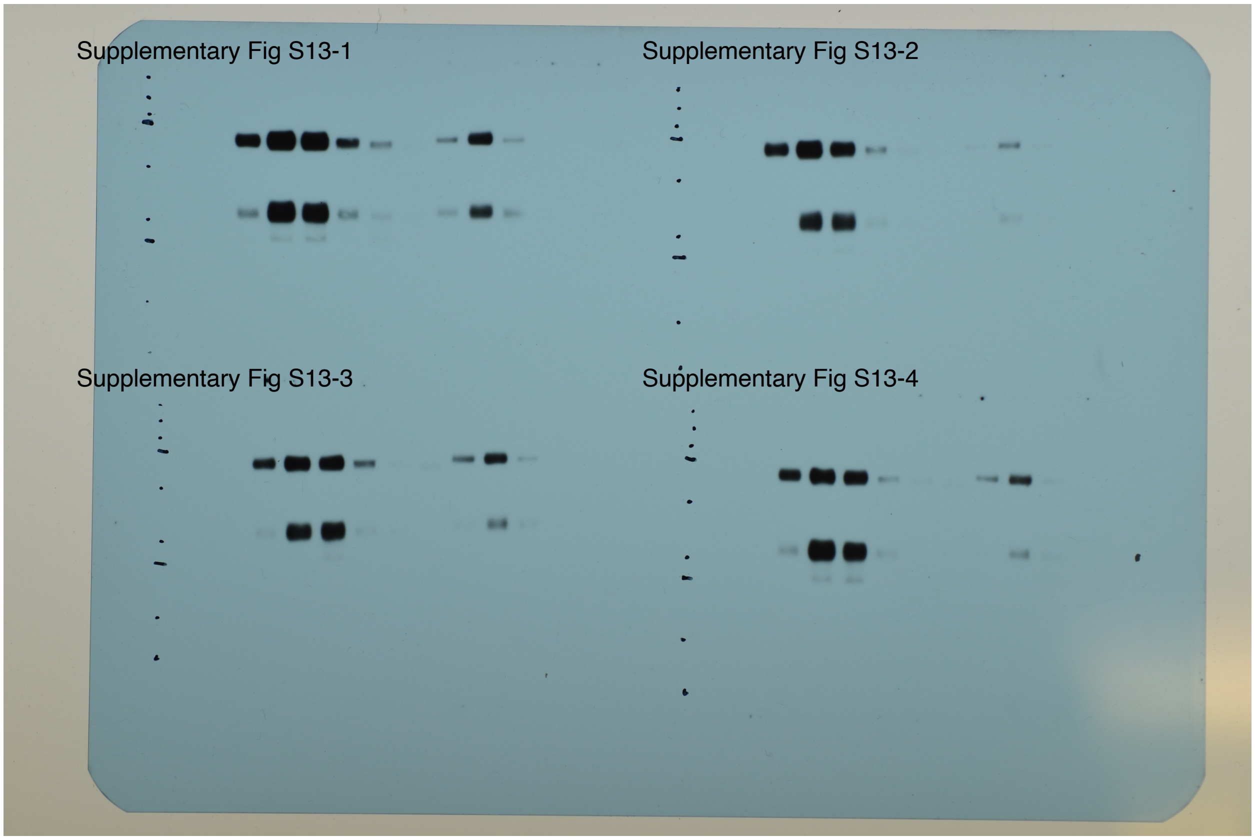

Supplementary Figure S13. Four replicates of the original immunoblots used to detect protein accumulation in the leaves of TVV3 lines. The gel of the blot used to generate Fig S13-1 was loaded with 5 µg of protein from each leaf. The gels of the blots used to generate Fig S13-2, S13-3, and S13-4 were loaded with 4 µg of protein from each leaf. In each blot a second line was used to generate the bands in lanes 10 through 15. Fig S13-1 is the original blot corresponding to Fig 3c.

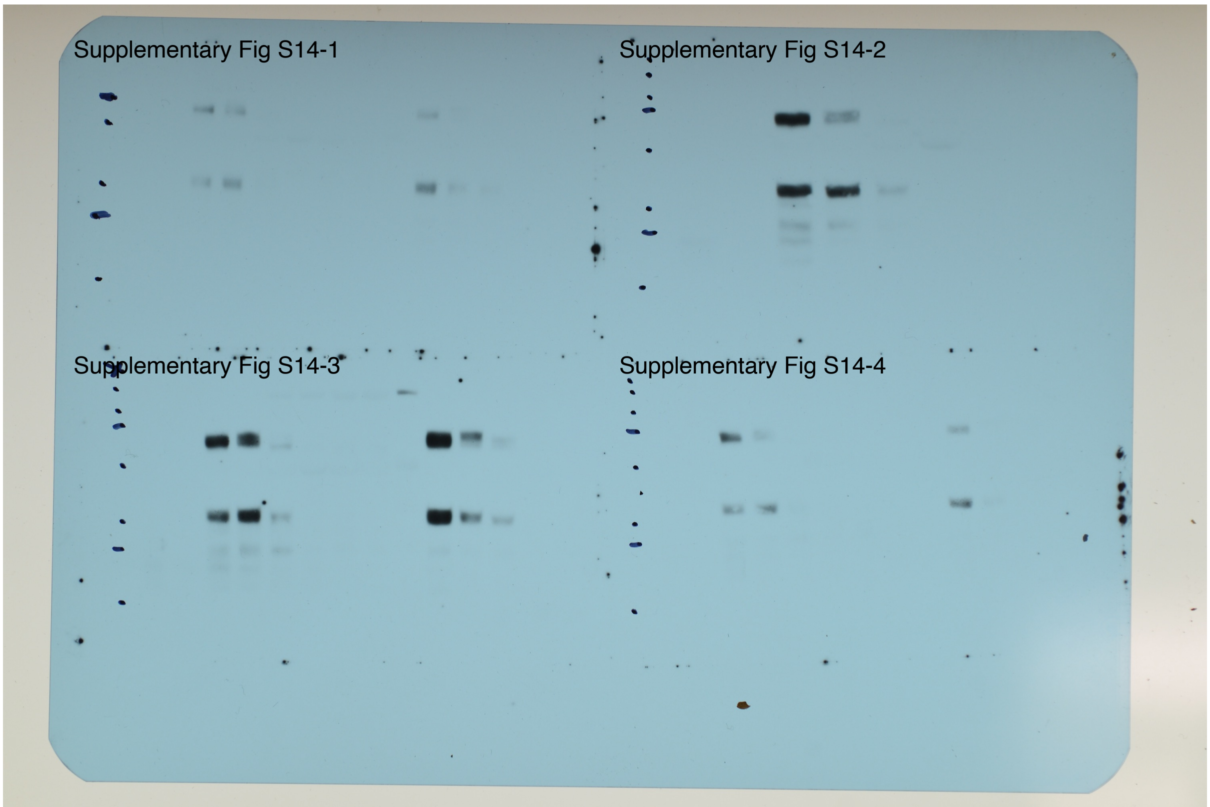

Supplementary Figure S14. Four replicates of the original immunoblots used to detect protein accumulation in the leaves of TVV4 lines. The gel of the blot used to generate Fig S14-1 was loaded with 10 µg of protein from each leaf. The gel of the blot used to generate Fig S14-2 was loaded with 40 µg of protein from each leaf. The gels of the blots used to generate Fig S14-3 and S14-4 were loaded with 20 µg of protein from each leaf. In blots S14-1, S14-3, and S14-4 a second line was used to generate the bands in lanes 11 through 15. Fig S14-3 is the original blot corresponding to Fig 3d.

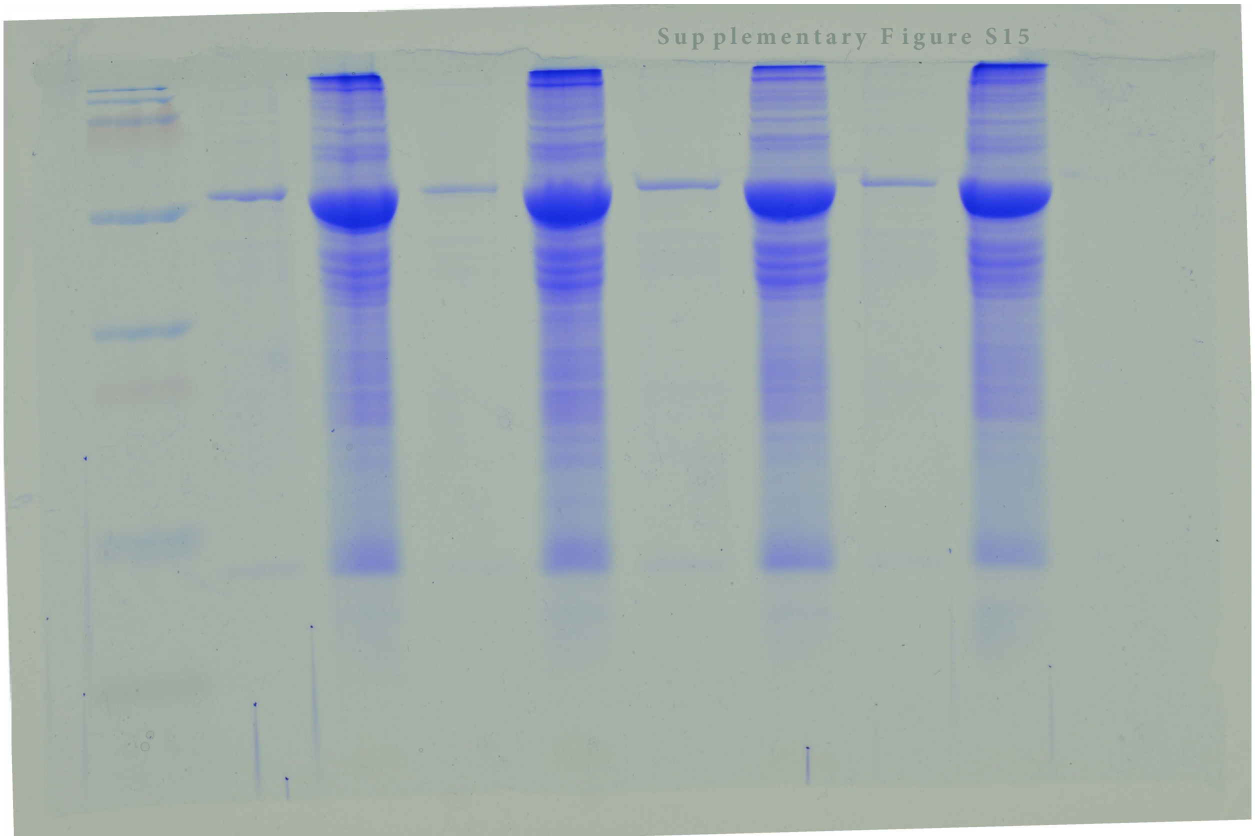

Supplementary Figure S15. Original Coomassie brilliant blue gel corresponding to Fig 3f.

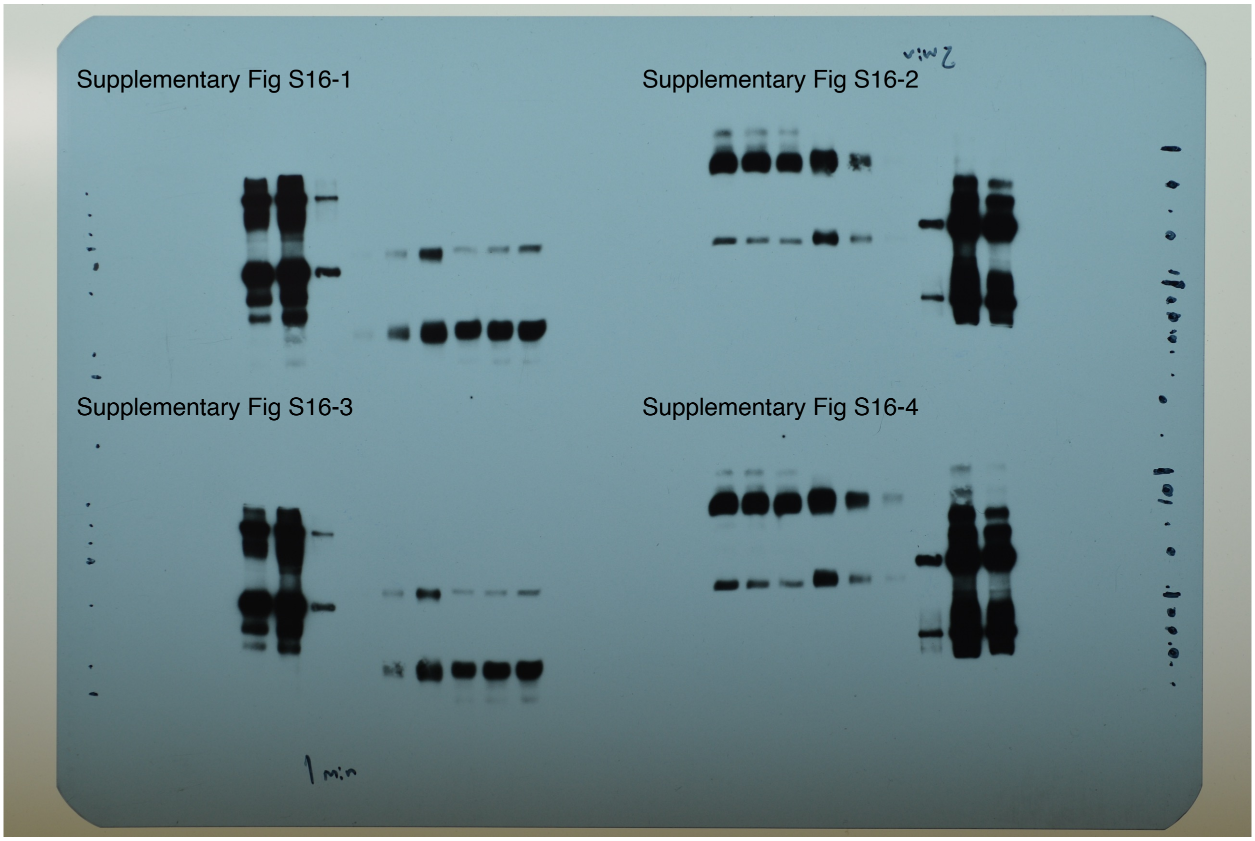

Supplementary Figure S16. Two replicates of the original immunoblots used to detect the forms of the Fc fusions post lyophilization in each line. The films of the blots used to generate Fig S16-1 and S16-3 were exposed for 1 minute. The films of the blots used to generate Fig S16-2 and S16-4 were exposed for 2 minutes and are rotated 180 degrees in the image. Fig S16-4 is the original blot corresponding to Fig 3g.

Supplementary Table S1. DNA sequence of TVV plastid transformation vectors. The vectors are based on pPRV1-II vector (GenBank Acc. No. U12809), a pUC119/pZS92 derivative, in which marker gene and gene of interest is cloned in the unique *Sca*I site (see Materials and Methods). TVV1, *aadA* with cMyc tag between *attB* and *attP* target sites and *trnP* gene. Vectors TVV2-TVV5, in addition to *aadA*, carry a P*rrn*L*atpH*-T*his*/*thr* cassette for the expression of GFP-Fc1 (TVV2), proIGF1-Fc1NC (TVV3), proIGF1-Fc1C (TVV4) and Myostatin-Fc1 (TVV5). The cassettes were cloned in as SacI-HindIII fragments. Annotated versions of the constructs are shown in Supplementary Figure S3.

>TVV1 targeting region cloned in ScaI site of pPRV1-II plasmid

CTTCCGCTTCCTCGCTCACTGACTCGCTGCGCTCGGTCGTTCGGCTGCGGCGAGCGGTATCAGCTCACTCAAAGGCGGTAATAC
GGTTATCCACAGAATCAGGGGATAACGCAGGAAAGAACATGTGAGCAAAAGGCCAGCAAAAGGCCAGGAACCGTAAAAAGG
CCGCGTTGCTGGCGTTTTTCCATAGGCTCCGCCCCCCTGACGAGCATCACAAAAATCGACGCTCAAGTCAGAGGTGGCGAAAC
CCGACAGGACTATAAAGATACCAGGCGTTTCCCCCTGGAAGCTCCCTCGTGCGCTCTCCTGTTCCGACCCTGCCGCTTACCGGA
TACCTGTCCGCCTTTCTCCCTTCGGGAAGCGTGGCGCTTTCTCATAGCTCACGCTGTAGGTATCTCAGTTCGGTGTAGGTCGTTC
GCTCCAAGCTGGGCTGTGTGCACGAACCCCCCGTTCAGCCCGACCGCTGCGCCTTATCCGGTAACTATCGTCTTGAGTCCAACC
CGGTAAGACACGACTTATCGCCACTGGCAGCAGCCACTGGTAACAGGATTAGCAGAGCGAGGTATGTAGGCGGTGCTACAGA
GTTCTTGAAGTGGTGGCCTAACTACGGCTACACTAGAAGGACAGTATTTGGTATCTGCGCTCTGCTGAAGCCAGTTACCTTCGG
AAAAAGAGTTGGTAGCTCTTGATCCGGCAAACAAACCACCGCTGGTAGCGGTGGTTTTTTTGTTTGCAAGCAGCAGATTACGC
GCAGAAAAAAAGGATCTCAAGAAGATCCTTTGATCTTTTCTACGGGGTCTGACGCTCAGTGGAACGAAAACTCACGTTAAGGG
ATTTTGGTCATGAGATTATCAAAAAGGATCTTCACCTAGATCCTTTTAAATTAAAAATGAAGTTTTAAATCAATCTAAAGTATA
TATGAGTAAACTTGGTCTGACAGTTACCAATGCTTAATCAGTGAGGCACCTATCTCAGCGATCTGTCTATTTCGTTCATCCATA
GTTGCCTGACTCCCCGTCGTGTAGATAACTACGATACGGGAGGGCTTACCATCTGGCCCCAGTGCTGCAATGATACCGCGAGA
CCCACGCTCACCGGCTCCAGATTTATCAGCAATAAACCAGCCAGCCGGAAGGGCCGAGCGCAGAAGTGGTCCTGCAACTTTAT
CCGCCTCCATCCAGTCTATTAATTGTTGCCGGGAAGCTAGAGTAAGTAGTTCGCCAGTTAATAGTTTGCGCAACGTTGTTGCCA
TTGCTACAGGCATCGTGGTGTCACGCTCGTCGTTTGGTATGGCTTCATTCAGCTCCGGTTCCCAACGATCAAGGCGAGTTACAT
GATCCCCCATGTTGTGCAAAAAAGCGGTTAGCTCCTTCGGTCCTCCGATCGTTGTCAGAAGTAAGTTGGCCGCAGTGTTATCAC
TCATGGTTATGGCAGCACTGCATAATTCTCTTACTGTCATGCCATCCGTAAGATGCTTTTCTGTGACTGGTGAGTAtTCAACCAA
GTCATTCTGAGAATAGTGTATGCGGCGACCGAGTTGCTCTTGCCCGGCGTCAATACGGGATAATACCGCGCCACATAGCAGAA
CTTTAAAAGTGCTCATCATTGGAAAACGTTCTTCGGGGCGAAAACTCTCAAGGATCTTACCGCTGTTGAGATCCAGTTCGATGT
AACCCACTCGTGCACCCAACTGATCTTCAGCATCTTTTACTTTCACCAGCGTTTCTGGGTGAGCAAAAACAGGAAGGCAAAAT
GCCGCAAAAAAGGGAATAAGGGCGACACGGAAATGTTGAATACTCATACTCTTCCTTTTTCAATATTATTGAAGCATTTATCA
GGGTTATTGTCTCATGAGCGGATACATATTTGAATGTATTTAGAAAAATAAACAAATAGGGGTTCCGCGCACATTTCCCCGAA
AAGTGCCACCTGACGTCTAAGAAACCATTATTATCATGACATTAACCTATAAAAATAGGCGTATCACGAGGCCCTTTCGTCTCG
CGCGTTTCGGTGATGACGGTGAAAACCTCTGACACATGCAGCTCCCGGAGACGGTCACAGCTTGTCTGTAAGCGGATGCCGGG
AGCAGACAAGCCCGTCAGGGCGCGTCAGCGGGTGTTGGCGGGTGTCGGGGCTGGCTTAACTATGCGGCATCAGAGCAGATTG
TACTGAGAGTGCACCATAAAATTGTAAACGTTAATATTTTGTTAAAATTCGCGTTAAATTTTTGTTAAATCAGCTCATTTTTTAA
CCAATAGGCCGAAATCGGCAAAATCCCTTATAAATCAAAAGAATAGCCCGAGATAGGGTTGAGTGTTGTTCCAGTTTGGAACA
AGAGTCCACTATTAAAGAACGTGGACTCCAACGTCAAAGGGCGAAAAACCGTCTATCAGGGCGATGGCCCACTACGTGAACC
ATCACCCAAATCAAGTTTTTTGGGGTCGAGGTGCCGTAAAGCACTAAATCGGAACCCTAAAGGGAGCCCCCGATTTAGAGCTT
GACGGGGAAAGCCGGCGAACGTGGCGAGAAAGGAAGGGAAGAAAGCGAAAGGAGCGGGCGCTAGGGCGCTGGCAAGTGTA
GCGGTCACGCTGCGCGTAACCACCACACCCGCCGCGCTTAATGCGCCGCTACAGGGCGCGTACTATGGTTGCTTTGACGTATG
CGGTGTGAAATACCGCACAGATGCGTAAGGAGAAAATACCGCATCAGGCGCCATTCGCCATTCAGGCTGCGCAACTGTTGGG
AAGGGCGATCGGTGCGGGCCTCTTCGCTATTACGCCAGCTGGCGAAAGGGGGATGTGCTGCAAGGCGATTAAGTTGGGTAAC
GCCAGGGTTTTCCCAGTCACGACGTTGTAAAACGACGGCCAGTGAATTAATTCACCGCCGTATGGCTGACCGGCGATTACTAG
CGATTCCGGCTTCATGCAGGCGAGTTGCAGCCTGCAATCCGAACTGAGGACGGGTTTTTGGGGTTAGCTCACCCTCGCGGGAT
CGCGACCCTTTGTCCCGGCCATTGTAGCACGTGTGTCGCCCAGGGCATAAGGGGCATGATGACTTGACGTCATCCTCACCTTCC
TCCGGCTTATCACCGGCAGTCTGTTCAGGGTTCCAAACTCAACGATGGCAACTAAACACGAGGGTTGCGCTCGTTGCGGGACT
TAACCCAACACCTTACGGCACGAGCTGACGACAGCCATGCACCACCTGTGTCCGCGTTCCCGAAGGCACCCCTCTCTTTCAAG
AGGATTCGCGGCATGTCAAGCCCTGGTAAGGTTCTTCGCTTTGCATCGAATTAAACCACATGCTCCACCGCTTGTGCGGGCCCC
CGTCAATTCCTTTGAGTTTCATTCTTGCGAACGTACTCCCCAGGCGGGATACTTAACGCGTTAGCTACAGCACTGCACGGGTCG
ATACGCACAGCGCCTAGTATCCATCGTTTACGGCTAGGACTACTGGGGTATCTAATCCCATTCGCTCCCCTAGCTTTCGTCTCTC
AGTGTCAGTGTCGGCCCAGCAGAGTGCTTTCGCCGTTGGTGTTCTTTCCGATCTCTACGCATTTCACCGCTCCACCGGAAATTC
CCTCTGCCCCTACCGTACTCCAGCTTGGTAGTTTCCACCGCCTGTCCAGGGTTGAGCCCTGGGATTTGACGGCGGACTTAAAAA
GCCACCTACAGACGCTTTACGCCCAATCATTCCGGATAACGCTTGCATCCTCTGTATTACCGCGGCTGCTGGCACAGAGTTAGC
CGATGCTTATTCCCCAGATACCGTCATTGCTTCTTCTCCGGGAAAAGAAGTTCACGACCCGTGGGCCTTCTACCTCCACGCGGC
ATTGCTCCGTCAGGCTTTCGCCCATTGCGGAAAATTCCCCACTGCTGCCTCCCGTAGGAGTCTGGGCCGTGTCTCAGTCCCAGT
GTGGCTGATCATCCTCTCGGACCAGCTACTGATCATCGCCTTGGTAAGCTATTGCCTCACCAACTAGCTAATCAGACGCGAGCC
CCTCCTCGGGCGGATTCCTCCTTTTGCTCCTCAGCCTACGGGGTATTAGCAGCCGTTTCCAGCTGTTGTTCCCCTCCCAAGGGCA
GGTTCTTACGCGTTACTCACCCGTCCGCCACTGGAAACACCACTTCCCGTCCGACTTGCATGTGTTAAGCATGCCGCCAGCGTT
CATCCTGAGCCAGGATCGAACTCTCCATGAGATTCATAGTTGCATTACTTATAGCTTCCTTGTTCGTAGACAAAGCGGATTCGG
AATTGTCTTTCATTCCAAGGCATAACTTGTATCCATGCGCTTCATATTCGCCCGGAGTTCGCTCCCAGAAATATAGCCATCCCT
GCCCCCTCACGTCAATCCCACGAGCCTCTTATCCATTCTCATTGAACGACGGCGGGGGAGCTTTCGAGGCCTCGAAATCCAACT

AGAAAAACTCACATTGGGCTTAGGGATAATCAGGCTCGAACTGATGACTTCCACCACGTCAAGGTGACACTCTACCGCTGAGT
TATATCCCTTCCCCGCCCCATCGAGAAATAGAACTGACTAATCCTAAGTCAAAGGGTCGAGAAACTCAACGCCACTATTCTTG
AACAACTTGGAGCCGGGCCTTCTTTTCGCACTATTACGGATATGAAAATAATGGTCAAAATCGGATTCAATTGTCAAAGTCCTT
TAATTAAGAATTCGAGCTCGGTACCCGGGGATCCTCTAGAGTCGACCTGCAGGCATGCAAGCTTAAGTCTTTACAATGACAAT
GGAAACCGATGTAAAGGGATGTAGCGCAGCTTGGTAGCGCGTTTGTTTTGGGTACAAAATGTCACAGGTTCAAATCCTGTCAT
CCCTATCCCTAACTTGTAGTTATCGTATCAGCAGTAACAATAGATGTGCCAGGGCGTGCCCTTGGGCTCCCCGGGCGCGTTGGG
TTGCGCTATATATATGAAAGAGTATACAATAATGATGTATTTGAGTTGTAGGGAGGGATTTATGGGGGAAGCGGTGATCGCCG
AAGTATCGACTCAACTATCAGAGGTAGTTGGCGTCATCGAGCGCCATCTCGAACCGACGTTGCTGGCCGTACATTTGTACGGC
TCCGCAGTGGATGGCGGCCTGAAGCCACACAGTGATATTGATTTGCTGGTTACGGTGACCGTAAGGCTTGATGAAACAACGCG
GCGAGCTTTGATCAACGACCTTTTGGAAACTTCGGCTTCCCCTGGAGAGAGCGAGATTCTCCGCGCTGTAGAAGTCACCATTGT
TGTGCACGACGACATCATTCCGTGGCGTTATCCAGCTAAGCGCGAACTGCAATTTGGAGAATGGCAGCGCAATGACATTCTTG
CAGGTATCTTCGAGCCAGCCACGATCGACATTGATCTGGCTATCTTGCTGACAAAAGCAAGAGAACATAGCGTTGCCTTGGTA
GGTCCAGCGGCGGAGGAACTCTTTGATCCGGTTCCTGAACAGGATCTATTTGAGGCGCTAAATGAAACCTTAACGCTATGGAA
CTCGCCGCCCGACTGGGCTGGCGATGAGCGAAATGTAGTGCTTACGTTGTCCCGCATTTGGTACAGCGCAGTAACCGGCAAAA
TCGCGCCGAAGGATGTCGCTGCCGACTGGGCAATGGAGCGCCTGCCGGCCCAGTATCAGCCCGTCATACTTGAAGCTAGACAG
GCTTATCTTGGACAAGAAGAAGATCGCTTGGCCTCGCGCGCAGATCAGTTGGAAGAATTTGTCCACTACGTGAAAGGCGAGAT
CACCAAGGTAGTGGGCAAAGAACAAAAACTCATTTCTGAAGAAGACTTGTGAGATCCTGGCCTAGTCTATAGGAGGTTTTGAA
AAGAAAGGAGCAATAATCATTTTCTTGTTCTATCAAGAGGGTGCTATTGCTCCTTTCTTTTTTTCTTTTTATTTATTTACTAGTAT
TTTACTTACATAGACTTTTTTGTTTACATTATAGAAAAAGAAGGAGAGGTTATTTTCTTGCATTTATTCATGCCCCAACTGGGGT
AACCTTTGAGTTCTCTCAGTTGGGGGGCGGCCGCAGGACTTTAACTGCCCCTATCGGAAATAGGATTGACTACCGATTCCGAA
GGAACTGGAGTTACATCTCTTTTCCATTCAAGAGTTCTTATGCGTTTCCACGCCCCTTTGAGACCCCGAAAAATGGACAAATTC
CTTTTCTTAGGAACACATACAAGATTCGTCACTACAAAAAGGATAATGGTAACCCTACCATTAACTACTTCATTTATGAATTTC
ATAGTAATAGAAATACATGTCCTACCGAGACAGAATTTGGAACTTGCTATCCTCTTGCCTAGCAGGCAAAGATTTACCTCCGTG
GAAAGGATGATTCATTCGGATCGACATGAGAGTCCAACTACATTGCCAGAATCCATGTTGTATATTTGAAAGAGGTTGACCTC
CTTGCTTCTCTCATGGTACACTCCTCTTCCCGCCGAGCCCCTTTTCTCCTCGGTCCACAGAGACAAAATGTAGGACTGGTGCCA
ACAATTCATCAGACTCACTAAGTCGGGATCACTAACTAATACTAATCTAATATAATAGTCTAATATATCTAATATAATAGAAA
ATACTAATATAATAGAAAAGAACTGTCTTTTCTGTATACTTTCCCCGGTTCCGTTGCTACCGCGGGCTTTACGCAATCGATCGG
ATTAGATAGATATCCCTTCAACATAGGTCATCGAAAGGATCTCGGAGACCCACCAAAGTACGAAAGCCAGGATCTTTCAGAAA
ACGGATTCCTATTCAAAGAGTGCATAACCGCATGGATAAGCTCACACTAACCCGTCAATTTGGGATCCAAATTCGAGATTTTCC
TTGGGAGGTATCGGGAAGGATTTGGAATGGAATAATATCGATTCATACAGAAGAAAAGGTTCTCTATTGATTCAAACACTGTA
CCTAACCTATGGGATAGGGATCGAGGAAGGGGAAAAACCGAAGATTTCACATGGTACTTTTATCAATCTGATTTATTTCGTAC
CTTTCGTTCAATGAGAAAATGGGTCAAATTCTACAGGATCAAACCTATGGGACTTAAGGAATGATATAAAAAAAAGAGAGGG
AAAATATTCATATTAAATAAATATGAAGTAGAAGAACCCAGATTCCAAATGAACAAATTCAAACTTGAAAAGGATCTTCCTTA
TTCTTGAAGAATGAGGGGCAAAGGGATTGATCAAGAAAGATCCTCTAGCTAGAGCTTGGCGTAATCATGGTCATAGCTGTTTC
CTGTGTGAAATTGTTATCCGCTCACAATTCCACACAACATACGAGCCGGAAGCATAAAGTGTAAAGCCTGGGGTGCCTAATGA
GTGAGCTAACTCACATTAATTGCGTTGCGCTCACTGCCCGCTTTCCAGTCGGGAAACCTGTCGTGCCAGCTGCATTAATGAATC
GGCCAACGCGCGGGGAGAGGCGGTTTGCGTATTGGGCGCT

>TVV2

GagctcGCTCCCCCGCCGTCGTTCAATGAGAATGGATAAGAGGCTCGTGGGATTGACGTGAGGGGGCAGGGATGGCTATATTTCT
GGGAGTTACTTCTACCCGATAGAGCTTAGAAGTTGGAAGTAATAATTTCTTGGTTGATTGTAtcCTTAACCATTTCTTTTTTTTTG
ACACGAGGAACTCATCATGGTCTCTAAGGGTGAAGAACTATTTACGGGTGTTGTACCTATTCTCGTCGAACTTGATGGGGATGT
CAACGGGCATAAGTTTAGCGTTTCCGGAGAGGGTGAGGGTGATGCTACGTACGGTAAGCTAACGCTTAAATTCATCTGCACGA
CAGGGAAACTGCCCGTTCCTTGGCCGACCCTTGTTACGACGTTGACCTATGGTGTTCAATGTTTCAGTAGATACCCCGATCAtAT
GAAGCAACATGATTTTTTTAAGAGTGCAATGCCCGAAGGTTACGTTCAAGAGAGGACAATCTTCTTTAAGGACGACGGTAACT
ACAAAACGCGCGCTGAAGTTAAATTTGAAGGTGATACGTTGGTGAACCGCATCGAGCTTAAGGGTATTGACTTCAAAGAGGAT
GGGAACATCTTAGGTCATAAACTTGAATATAACTATAATTCGCATAACGTATATATTATGGCTGACAAACAAAAAAACGGTAT
TAAAGTTAATTTTAAAATTCGACATAACATCGAAGATGGTTCTGTTCAATTGGCCGATCATTATCAACAAAACACGCCTATCGG
GGACGGTCCAGTGCTATTACCCGACAATCACTATCTTTCAACCCAATCTGCTCTCTCTAAAGACCCCAATGAAAAACGAGACC
ACATGGTCTTGCTAGAATTTGTTACtGCaGCGGGTATCACGCTTGGGATGGACGAACTGTATAAAGGGGGGGGTGGTTGCAAGC
CCTGTATTTGTACGGTaCCGGAAGTATCTTCTGTGTTCATTTTTCCCCCTAAACCAAAGGATGTCTTGACGATTACGCTTACCCC
GAAAGTTACGTGTGTTGTTGTTGACATCAGTAAAGATGACCCTGAAGTTCAATTTTCTTGGTTCGTTGACGATGTCGAAGTTCA
TACGGCTCAAACGCAACCTAGAGAGGAGCAATTTAATTCCACCTTTCGATCAGTCAGTGAACTCCCCATCATGCACCAAGACT
GGCTTAACGGGAAAGAGTTCAAATGCCGCGTAAACTCAGCAGCTTTCCCCGCCCCTATCGAAAAGACCATCTCTAAAACGAAG
GGTAGGCCCAAAGCTCCACAAGTTTATACGATCCCCCCTCCCAAAGAGCAAATGGCTAAAGATAAAGTCTCGTTAACGTGCAT
GATCACAGATTTTTTTCCCGAGGACATCACGGTTGAGTGGCAATGGAATGGTCAACCTGCTGAAAACTATAAAAACACGCAAC
CTATTATGGATACGGACGGGTCGTATTTTGTGTACTCTAAGCTAAATGTaCAAAAGTCCAACTGGGAAGCGGGTAACACGTTTA
CATGTTCTGTACTGCACGAGGGTTTGCATAACCATCATACAGAGAAATCACTTAGCCATAGTCCAGGGAAATAAatatagACGCGT
TCAATTTAAACACCACCATCATCACCATCATCCTGACTAGaCTTTCAGGCGATGTGTGCTGGAAGACATTCgGATCTTCCAGTGG
TGCATGAACGCATGAGAAAGCCCCCGGAAGATCATCTTCCGGGGGCTTTTTTTTGGCGCGTGACGCGTACAGGAAACACAGA
AAAAAGCCCGCACCTGACAGTGCGGGCTTTTTTTTTCGACCAAAGGTAACGAGGTAACAACCATGCGaagctt

>TVV3

GagctcGCTCCCCCGCCGTCGTTCAATGAGAATGGATAAGAGGCTCGTGGGATTGACGTGAGGGGGCAGGGATGGCTATATTTCT
GGGAGTTACTTCTACCCGATAGAGCTTAGAAGTTGGAAGTAATAATTTCTTGGTTGATTGTAtcCTTAACCATTTCTTTTTTTTTG
ACACGAGGAACTCATCATGACCCTTTGTGGTGCTGAACTGGTTGACGCTTTACAATTTGTCTGCGGTCCCAGGGGTTTTATTT
CAACAAGCCCACGGGTTATGGTAGTTCTATTCGAAGGGCACCACAAACGGGGATCGTGGATGAGTGTTGTTTTCGCTCGTGCG
ATCTTCGACGCCTAGAGATGTATTGTGCTCCCTTGAAACCGACGGGGGCCGCGCGCTCAATCGCTGCTCAAGCTCACACAGAC
ATGCCTAAAACGCAAAAAGAGGTTCATCTCAAAAATACGTCTCGCGGTAGCGCTGGTAACAAGACGTACCGAATGgggggggtG

GTTGCAAGCCCTGTATTTGTACGGTaCCGGAAGTATCTTCTGTGTTCATTTTTCCCCCTAAACCAAAGGATGTCTTGACGATTAC
GCTTACCCCGAAAGTTACGTGTGTTGTTGTTGACATCAGTAAAGATGACCCTGAAGTTCAATTTTCTTGGTTCGTTGACGATGT
CGAAGTTCATACGGCTCAAACGCAACCTAGAGAGGAGCAATTTAATTCCACCTTTCGATCAGTCAGTGAACTCCCCATCATGC
ACCAAGACTGGCTTAACGGGAAAGAGTTCAAATGCCGCGTAAACTCAGCAGCTTTCCCCGCCCCTATCGAAAAGACCATCTCT
AAAACGAAGGGTAGGCCCAAAGCTCCACAAGTTTATACGATCCCCCCTCCCAAAGAGCAAATGGCTAAAGATAAAGTCTCGTT
AACGTGCATGATCACAGATTTTTTTCCCGAGGACATCACGGTTGAGTGGCAATGGAATGGTCAACCTGCTGAAAACTATAAAA
ACACGCAACCTATTATGGATACGGACGGGTCGTATTTTGTGTACTCTAAGCTAAATGTaCAAAAGTCCAACTGGGAAGCGGGT
AACACGTTTACATGTTCTGTACTGCACGAGGGTTTGCATAACCATCATACAGAGAAATCACTTAGCCATAGTCCAGGGAAATA
AatatagACGCGTTCAATTTAAACACCACCATCATCACCATCATCCTGACTAGaCTTTCAGGCGATGTGTGCTGGAAGACATTCgGA
TCTTCCAGTGGTGCATGAACGCATGAGAAAGCCCCCGGAAGATCATCTTCCGGGGGCTTTTTTTTTGGCGCGTGACGCGTACAG
GAAACACAGAAAAAAGCCCGCACCTGACAGTGCGGGCTTTTTTTTTCGACCAAAGGTAACGAGGTAACAACCATGCGaagctt

TVV4

GagctcGCTCCCCCGCCGTCGTTCAATGAGAATGGATAAGAGGCTCGTGGGATTGACGTGAGGGGGCAGGGATGGCTATATTTCT
GGGAGTTACTTCTACCCGATAGAGCTTAGAAGTTGGAAGTAATAATTTCTTGGTTGATTGTAtcCTTAACCATTTCTTTTTTTTTG
ACACGAGGAACTCATCATGGGGCCTGAAACCCTTTGTGGTGCTGAACTGGTTGACGCTTTACAATTTGTCTGCGGTCCCAGGG
GTTTTTATTTCAACAAGCCCACGGGTTATGGTAGTTCTATTCGAAGGGCACCACAAACGGGGATCGTGGATGAGTGTTGTTTTC
GCTCGTGCGATCTTCGACGCCTAGAGATGTATTGTGCTCCCTTGAAACCGACGGGGGCCGCGCGCTCAATCGCTGCTCAAGCTC
ACACAGACATGCCTAAAACGCAAAAAGAGGTTCATCTCAAAAATACGTCTCGCGGTAGCGCTGGTAACAAGACGTACCGAAT
GGGGCGATCCAGACGAGGTCGATCTCGAAGAGGTTGCAAGCCCTGTATTTGTACGGTaCCGGAAGTATCTTCTGTGTTCATTTT
TCCCCCTAAACCAAAGGATGTCTTGACGATTACGCTTACCCCGAAAGTTACGTGTGTTGTTGTTGACATCAGTAAAGATGACCC
TGAAGTTCAATTTTCTTGGTTCGTTGACGATGTCGAAGTTCATACGGCTCAAACGCAACCTAGAGAGGAGCAATTTAATTCCAC
CTTTCGATCAGTCAGTGAACTCCCCATCATGCACCAAGACTGGCTTAACGGGAAAGAGTTCAAATGCCGCGTAAACTCAGCAG
CTTTCCCCGCCCCTATCGAAAAGACCATCTCTAAAACGAAGGGTAGGCCCAAAGCTCCACAAGTTTATACGATCCCCCCTCCC
AAAGAGCAAATGGCTAAAGATAAAGTCTCGTTAACGTGCATGATCACAGATTTTTTTCCCGAGGACATCACGGTTGAGTGGCA
ATGGAATGGTCAACCTGCTGAAAACTATAAAAACACGCAACCTATTATGGATACGGACGGGTCGTATTTTGTGTACTCTAAGC
TAAATGTaCAAAAGTCCAACTGGGAAGCGGGTAACACGTTTACATGTTCTGTACTGCACGAGGGTTTGCATAACCATCATACA
GAGAAATCACTTAGCCATAGTCCAGGGAAATAAatatagACGCGTTCAATTTAAACACCACCATCATCACCATCATCCTGACTAGa
CTTTCAGGCGATGTGTGCTGGAAGACATTCgGATCTTCCAGTGGTGCATGAACGCATGAGAAAGCCCCCGGAAGATCATCTTCC
GGGGGCTTTTTTTTTGGCGCGTGACGCGTACAGGAAACACAGAAAAAAGCCCGCACCTGACAGTGCGGGCTTTTTTTTTCGAC
CAAAGGTAACGAGGTAACAACCATGCGaagctt

TVV5

GagctcGCTCCCCCGCCGTCGTTCAATGAGAATGGATAAGAGGCTCGTGGGATTGACGTGAGGGGGCAGGGATGGCTATATTTCT
GGGAGTTACTTCTACCCGATAGAGCTTAGAAGTTGGAAGTAATAATTTCTTGGTTGATTGTAtcCTTAACCATTTCTTTTTTTTTG
ACACGAGGAACTCATCATGAACGAAGGGTCTGAAAGGGAGGAGAATGTTGAAAAGGAGGGTTTGTGTAACGCTTGTGCTTGG
CGACAAAACACGCGATATAGTAGAATTGAAGCTATTAAAATTCAAATCCTATCGAAGCTGCGACTTGAAACGGCTCCGAACAT
CTCCAAAGACGCCATTCGACAGCTGCTACCTCGCGCCCCTCCACTACGCGAACTCATTGACCAATACGATGTGCAAAGGGCTG
ACTCGTCAGATGGGTCTTTGGAAGACGACGATTATCACGCTACGACGGAAACAATCATCACGATGCCCACCGAATCAGATTTT
CTTATGCAAGCAGATGGTAAACCTAAATGCTGCTTTTTTAAATTTTCTAGTAAAATCCAATATAACAAAGTAGTTAAAGCTCAA
CTTTGGATTTACTTACGCCCTGTCAAAACACCGACCACGGTATTCGTTCAAATCCTTAGACTAATCAAACCCATGAAAGATGGG
ACGCGATATACCGGTATCCGCTCTTTGAAATTAGACATGAGCCCCGGTACGGGGATCTGGCAATCCATTGATGTTAAAACGGT
TCTCCAAAACTGGCTTAAGCAACCCGAATCTAATCTTGGTATCGAGATTAAGGCTCTTGACGAAAACGGTCATGACCTGGCGG
TGACATTCCCTGGTCCCGGTGAAGACGGTTTGAATCCATTTctcGAGGTCAAGGTTACGGATACGCCGAAGGGGCTTGATGGTG
GTTGCAAGCCCTGTATTTGTACGGTaCCGGAAGTATCTTCTGTGTTCATTTTTCCCCCTAAACCAAAGGATGTCTTGACGATTAC
GCTTACCCCGAAAGTTACGTGTGTTGTTGTTGACATCAGTAAAGATGACCCTGAAGTTCAATTTTCTTGGTTCGTTGACGATGT
CGAAGTTCATACGGCTCAAACGCAACCTAGAGAGGAGCAATTTAATTCCACCTTTCGATCAGTCAGTGAACTCCCCATCATGC
ACCAAGACTGGCTTAACGGGAAAGAGTTCAAATGCCGCGTAAACTCAGCAGCTTTCCCCGCCCCTATCGAAAAGACCATCTCT
AAAACGAAGGGTAGGCCCAAAGCTCCACAAGTTTATACGATCCCCCCTCCCAAAGAGCAAATGGCTAAAGATAAAGTCTCGTT
AACGTGCATGATCACAGATTTTTTTCCCGAGGACATCACGGTTGAGTGGCAATGGAATGGTCAACCTGCTGAAAACTATAAAA
ACACGCAACCTATTATGGATACGGACGGGTCGTATTTTGTGTACTCTAAGCTAAATGTaCAAAAGTCCAACTGGGAAGCGGGT
AACACGTTTACATGTTCTGTACTGCACGAGGGTTTGCATAACCATCATACAGAGAAATCACTTAGCCATAGTCCAGGGAAATA
AatatagACGCGTTCAATTTAAACACCACCATCATCACCATCATCCTGACTAGaCTTTCAGGCGATGTGTGCTGGAAGACATTCgGA
TCTTCCAGTGGTGCATGAACGCATGAGAAAGCCCCCGGAAGATCATCTTCCGGGGGCTTTTTTTTTGGCGCGTGACGCGTACAG
GAAACACAGAAAAAAGCCCGCACCTGACAGTGCGGGCTTTTTTTTTCGACCAAAGGTAACGAGGTAACAACCATGCGaagctt
